# Mathematical modeling of HIV-HCV co-infection model: Impact of parameters on reproduction number

**DOI:** 10.12688/f1000research.124555.2

**Published:** 2022-12-19

**Authors:** Oluwakemi E. Abiodun, Olukayode Adebimpe, James A. Ndako, Olajumoke Oludoun, Benedicta Aladeitan, Michael Adeniyi

**Affiliations:** 1 Physical Sciences, Landmark University, Omu Aran, State, 251101, Nigeria; 2 Mathematics and Statistics, First Technical University, Ibadan, Oyo, Nigeria; 3 Mathematics and Statistics, Lagos State Polytechnic, Lagos, Lagos, Nigeria

**Keywords:** Mathematical model, HIV/AIDS, HCV, infection-free equilibrium, unawareness, awareness, endemic equilibrium, next generation matrix, basic reproduction number, stability.

## Abstract

**Background: **Hepatitis C Virus (HCV) and Human Immunodeficiency Virus (HIV) are both classified as blood-borne viruses since they are transmitted through contact with contaminated blood. Approximately 1.3 million of the 2.75 million global HIV/HCV carriers are people who inject drugs (PWID). HIV co-infection has a harmful effect on the progression of HCV, resulting in greater rates of HCV persistence after acute infection, higher viral levels, and accelerated progression of liver fibrosis and end-stage liver disease. In this study, we developed and investigated a mathematical model for the dynamical behavior of HIV/AIDS and HCV co-infection, which includes therapy for both diseases, vertical transmission in HIV cases, unawareness and awareness of HIV infection, inefficient HIV treatment follow-up, and efficient condom use.

**Methods: **Positivity and boundedness of the model under investigation were established using well-known theorems. The equilibria were demonstrated by bringing all differential equations to zero. The associative reproduction numbers for mono-infected and dual-infected models were calculated using the next-generation matrix approach. The local and global stabilities of the models were validated using the linearization and comparison theorem and the negative criterion techniques of bendixson and dulac, respectively.

**Results: **The growing prevalence of HIV treatment dropout in each compartment of the HIV model led to a reduction in HIV on treatment compartments while other compartments exhibited an increase in populations **. **In dually infected patients, treating HCV first reduces co-infection reproduction number *R*_*ech*_, which reduces liver cancer risk.

**Conclusions: **From the model's results, we infer various steps (such as: campaigns to warn individuals about the consequences of having multiple sexual partners; distributing more condoms to individuals; continuing treatment for chronic HCV and AIDS) that policymakers could take to reduce the number of mono-infected and co-infected individuals.

## Introduction

Emerging and reemerging infectious illnesses are of public health importance, and mathematics has traditionally been employed to acquire a realistic understanding of the transmission dynamics and control of these diseases. Both Hepatitis C Virus (HCV) and Human Immunodeficiency Virus (HIV) are considered blood-borne viruses because they are spread through contact with the blood of an infected individual. ^1^ In 2017, 2.3 million people living with HIV were simultaneously infected with HCV, according to the World Health Organization (WHO, 2016). Infectious diseases like HIV and HCV have become critical problems in public health around the world. Africa and South and East Asia bear the heaviest brunt of these co-infections (WHO, 2017). Co-infection with HIV and other diseases usually poses greater dangers and has more dire outcomes for individuals. When HIV is present alongside HCV, the viral infection advances much more quickly in the latter. If the CD4 cells is less than 200 cells/mm ^3^, the risk of severe liver injury increases. ^2^ Hepatocellular carcinoma, liver cirrhosis, and liver-related mortality are also more likely to occur. ^3^ The international community agrees that strong leadership in the form of well-thought-out programs and policies that focus on prevention, early diagnosis, therapies that respect patients' rights, and high-quality, universally accessible health care is needed to stop the spread of HIV. Concerning co-infection, there have been reports of effective HCV drug combinations in treating HIV-positive and HCV-positive people. Furthermore, HIV can be treated successfully in the majority of people with HCV. ^4^ New antiviral medications have the potential to treat HCV in persons who are HIV-positive and infected with HIV, but additional research is needed to prove their effectiveness.

There are about 40 million people living with HIV (PLHIV) in the world right now. UNAIDS, the United Nations Program on HIV/AIDS, estimates that in 2020, more than one person every minute would die from an AIDS-related illness. ^5^ HIV and HCV can be spread in many ways, such as through injections, sexual contact, and being passed down from parent to child. ^6^ People with HIV often also have Hepatitis B virus (HBV) and/or HCV. ^7^^,^^8^ One of the main reasons people with HIV die is because of liver disease. ^9^^,^^10^ There are over 2 million PLHIV on a global scale who are living with HBV or HCV. ^1^^,^^7^^,^^8^ Bi-directional effects explain why people who have HIV who also have HBV and/or HCV have a greater risk of becoming sick and die. HIV patients with HBV and/or HCV quickly develop AIDS, ^11^ and antiretroviral drugs are more harmful to the liver. ^12^^–^^14^ On the other hand, when PLHIV change their immune response, it leads to less HCV viral clearance, reactivation, and replication in co-infected individuals. Aspartate aminotransferase, alanine aminotransferase, and alkaline phosphatase levels rise as a result, and chronic liver disease complications like cirrhosis, hepatic decompensation, and hepatocellular carcinoma as well as a higher death rate ^15^^–^^17^ progress more quickly. People living with HIV who also have HBV, HCV, or both have a greater risk of infection transmission. However, there has been a relatively little deterministic study of HCV chronic infection co-infected with HIV. For instance, Ref. 18, introduced and analyzed a deterministic model for HCV and HIV co-infection. Focusing on HCV and HIV co-infection, they hope to better understand the long- and short-term dynamics of both diseases and develop methods for forecasting whether HCV and HIV will eventually become extinct or remain a persistent problem. To ascertain the effect of treatment on each disease’s dynamics ^19^ built and investigated a mathematical model of the co-dynamics of HCV and HIV/AIDS. The equilibria (disease-free and endemic) are described under which they are both locally and globally asymptotically stable. Similarly, Ref. 20, investigated the mathematical model of co-infection with HIV and HCV. In the case of HIV, the innovation of their strategy is the incorporation of therapy for both infections as well as how it is passed from mother to kid. ^21^ Constructed a mathematical model of HCV/HIV co-infection within the host by modifying a model of HCV mono-infection that had previously been published to include an immune system component in infection clearance. They then combined a decline in immunological function with an increase in HIV viral load to examine the impact of HIV co-infection on spontaneous HCV clearance and sustained virologic response (SVR). Additionally, using mathematics, ^22^ developed a new co-infection model for the human immunodeficiency virus (HIV) and hepatitis C virus (HCV). Examining therapy for both diseases, prevention, diagnosis, screening, HIV education and awareness, condom use, and largely employing numerical simulations. In, Ref. 23, constructed two ODE models at the population level to mimic the progression of HCV and HIV among PWID. Both deterministic and stochastic solutions were used to solve the models describing HCV and HIV parenteral transmission. Additionally, several deterministic models that are relevant to our work have been suggested and examined in Refs. 24– 29.

## The HIV and HCV co-infection model

The paradigm of co-infection between HIV and HCV is described in this section.

We determine overall and submodel reproduction rates (HIV only and HCV only models). We investigate the global and full model disease-free equilibrium local stability. We determine the reproduction number's, sensitivity indices to important model parameters. Simulation diagrams created using Runge-kutta order four embedded in maple 2020.1 software and contour plots created using maple 2020.1, help understand the model's dynamics.

### Full model description

The mathematical model that will be considered and investigated is divided into (15) different groups, namely, the susceptible populace for both HIV and HCV $S\left(t\right)$ , the HIV-infected unaware $H_{U}\left(t\right)$ , the HIV-infected aware, $H_{A}\left(t\right)$ , HIV on treatment $H_{T}\left(t\right)$ , the AIDS populace aware and on treatment $A_{A}\left(t\right)$ , acutely infected $I_{c}\left(t\right)$ and chronically infected $C_{c}\left(t\right)$ infected HCV, HIV-unaware co-infected with acute and chronic HCV ( $H_{uI}\left(t\right)\;\text{and}\;H_{\mathrm{uC}}\left(t\right)),$ HIV-aware co-infected with acute and chronic HCV ( $H_{\mathrm{AI}}\left(t\right)\;\text{and}\;H_{\mathrm{AC}}\left(t\right)),$ HIV-positive individuals receiving treatment for HIV who are co-infected with acute and chronic HCV ( $H_{\mathrm{TI}}\left(t\right)\;\text{and}\;H_{\mathrm{TC}}\left(t\right)),$ HIV-positive individuals in stage-IV co-infected with acute and chronic HCV $\;\left(A_{\mathrm{AI}}\left(t\right)\;\text{and}\;A_{\mathrm{AC}}\left(t\right)\right)$ .

The overall population at time t, represented by $N\left(t\right),$ is classified into the 15 classes/subgroups listed in Tables of Nomenclature, each of which corresponds to a different epidemiological status.

$$N\left(t\right)=S\left(t\right)+H_{U}\left(t\right)+H_{A}\left(t\right)+H_{T}\left(t\right)+A_{A}\left(t\right)+I_{c}\left(t\right)+C_{c}\left(t\right)+H_{uI}\left(t\right)+H_{\mathrm{AI}}\left(t\right)+H_{\mathrm{TI}}\left(t\right)+H_{\mathrm{UC}}\left(t\right)+H_{\mathrm{AC}}\left(t\right)+H_{\mathrm{TC}}\left(t\right)+A_{\mathrm{AI}}\left(t\right)+A_{\mathrm{AC}}\left(t\right)$$ (1)

**Figure 1.**
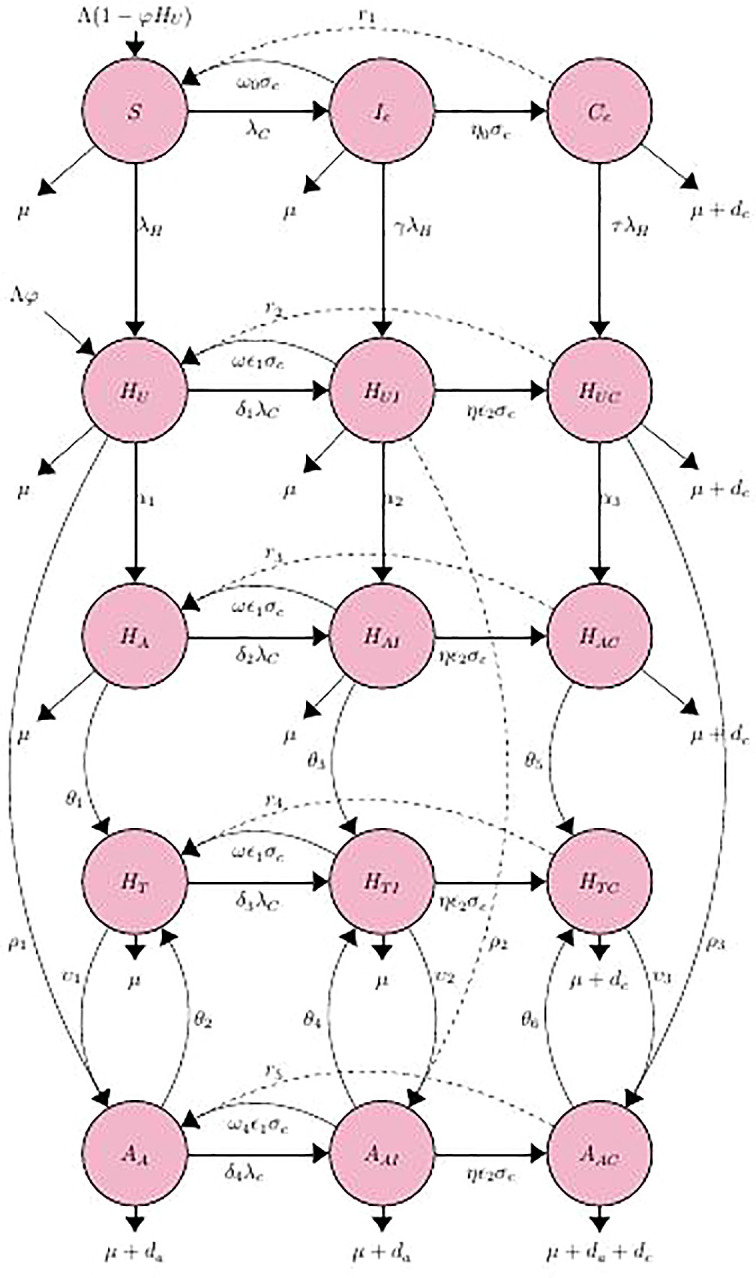
The compartmental flow diagram of the HIV-HCV co-infection.

In Figure 1, the epidemiology of co-infection with HIV and HCV is depicted schematically. The many compartments (circles) symbolize the various disease phases, and the arrows depict how people progress from one phase to the next. At time *t*, susceptible individuals *S* are assumed to enter the population at a constant rate, $\left(1-\varphi H_{u}\right)\Lambda .$ Some newborns acquire HIV at parturition and are subsequently enrolled directly into the infectious class, $H_{U}$ where φ , is the rate of newborn HIV infection and Λ is the rate of recruitment through immigration or emigration. Individuals in all classes die at a consistent natural mortality rate, μ. Individuals with AIDS $\left(A_{A},A_{\mathrm{AI}},A_{\mathrm{AC}}\right)$ have an extra death rate owing to AIDS, $d_{a}.$ We assume that HIV-infected people who are receiving treatment do not spread the virus. ^30^^,^^31^ Despite the complexity of disease co-dynamics, we will make the simple assumption that co-infected and mono-infected people can only transmit one of the two diseases—HIV or HCV—at a time. Individual S , who is susceptible to HIV infection, is at risk of acquiring HIV infection at a rate of $\;\lambda_{H}$ , (force of infection related to HIV) when in contact with the $H_{U},\;H_{A},\;\text{and}\;A_{A}$ populations, where

$$\lambda_{H}=c_{h}\left(1-\psi \xi \right)b_{h}\frac{H_{U}\left(t\right)+A_{A}\left(t\right)+\kappa_{1}\left(H_{uI}\left(t\right)+H_{\mathrm{uC}}\left(t\right)\right)}{N}$$ (2)

The parameter $b_{h}$ is the chance that a person will get HIV from a contact, and the parameter, the average annual number of sexual partners for someone at risk of contracting HIV is $c_{h}$ . To highlight the usage of condoms as a crucial prevention measure, we presume that $\psi \xi \in \left[0,1\right]\;$ indicates the degree of condom protection. If ξ=0, condom use offers no protection, ξ=1 denotes perfect protection, where ψ is the use of a condom.

When compared to persons who are only infected with HIV, the relative infectiousness of persons who are acutely infected with HCV and unaware of their HIV infection $\left(H_{\mathrm{UI}}\right)$ and individuals who are chronically infected with HCV and AIDS $\left(A_{\mathrm{Ac}}\right)$ , is accounted for by the parameters $\;\kappa_{1}>1$ . We make the assumption that persons who are co-infected are approximately three times more infectious than individuals who just have one infection. ^32^^,^^33^ HIV unaware class, $H_{U},\;H_{\mathrm{UI}},\;H_{\mathrm{UC}}$ singly and dually infected with HCV advances to HIV diagnosed class $H_{A},H_{\mathrm{AI}},H_{\mathrm{AC}}$ after testing at a rate, $\alpha_{1},\alpha_{2},\alpha_{3}\;$ and those in aware HIV was enrolled on therapy at the rate $\theta_{1},\theta_{3},\theta_{5}$ in class $H_{T},H_{\mathrm{TI}},H_{\mathrm{TC}}.$ Nevertheless, some individuals who were placed on HIV treatment default from or drop out of the Highly Active Antiretroviral Therapy (HAART) ^25^ after which they develop AIDS due to drug resistance and progress to class $A_{A},A_{\mathrm{AI}},A_{\mathrm{AC}}$ at a rate $\upsilon_{1},\upsilon_{2},\upsilon_{3}.$ People with HIV and HCV who don't know their HIV status, $H_{U},H_{\mathrm{UI}},H_{\mathrm{UC}}$ and didn't get tested move to the AIDS class $A_{A},A_{\mathrm{AI}},A_{\mathrm{AC}},$ at a rate $\rho_{1},\rho_{2},\rho_{3}$ . People with AIDS symptoms singly and dually infected with HCV are given treatment at a rate of $\theta_{2},\theta_{4},\theta_{6}$ respectively. AIDS infected can respond well to treatment and return to $H_{T},H_{\mathrm{TI}},H_{\mathrm{TC}}$ ^18^^,^^20^ and die because of AIDS at an incidence $d_{a}$ .

Susceptible people get HCV infection from people in the $I_{c},C_{c},H_{\mathrm{UI}},H_{\mathrm{UC}}$ at a rate of $\lambda_{C}$ where $\lambda_{C}\;$ is the risk of getting HCV, which is given by

$$\lambda_{C}=c\left(1-\psi \xi \right)b_{c}\frac{I_{c}\left(t\right)+C_{c}\left(t\right)+\kappa_{2}\left(H_{uI}\left(t\right)+H_{\mathrm{uC}}\left(t\right)\right)}{N}$$ (3)

To simulate the reality that individuals who are dually infected are more infectious than the mono-infected, we use the notation $\;\kappa_{2}>1$ , where $b_{c}\;$ the likelihood that contact will result in HCV infection. ^19^^,^^34^^,^^35^

People who are only infected with HIV $\left(H_{U},H_{A},H_{T},\text{and}\;A_{a}\right)$ acquired HCV at a rate $\left(\delta_{1}\lambda_{c},\delta_{2}\lambda_{c},\delta_{3}\lambda_{c}\right)$ and moved to classes $\;\left(H_{\mathrm{UI}},H_{\mathrm{AI}},H_{\mathrm{TI}},A_{\mathrm{AI}}\right)$ , an increased risk of HCV acquisition is accounted for by the modification parameter $\;\delta_{1},\delta_{2},\delta_{3}>1$ . HCV-only infected people $\left(\mathrm{Ic},C_{c}\right)$ are more likely to obtain HIV $\left(H_{\mathrm{uI}},H_{\mathrm{uC}}\right)$ than people who are only infected with HCV at a rate ${\gamma \lambda }_{H},{\tau \lambda }_{H}$ where γ,τ>1 translates to an increased chance of contracting HIV for people whose immune systems are weakened by HCV.

HIV and AIDS patients, dually infected with the acute HCV $H_{\mathrm{UI}},H_{\mathrm{TI}},A_{\mathrm{AI}}$ at a rate η, becomes chronically infected and are treated for chronic HCV epidemic at $r_{i},i=1,2,3,4,5$ while the remaining populace, ω spontaneously clear the virus to return to susceptible class S . We then assume that an individual whose immune system helps in clearing the virus can become re-infected at rate $\lambda_{C}$ if expose or engage in risk behaviors such as injection drug use, ^33^ drinking alcohol, ^36^ multiple sex partners and sex between two men ^37^ since the clearance does not confer permanent immunity. ^38^

An HCV-positive person stays acutely infected for an average of $1/\sigma_{c}$ days. Since newer combinations of direct-acting antivirals (DAAs) have showed high cure rates of 90%-95% in phase II and III clinical trials, we did not take treatment failure for chronic HCV carriers into account. However, researchers are beginning to report sporadic incidences of treatment failure in HCV. ^33^^,^^39^^,^^40^

In people with HIV and HCV co-infection, little is known regarding the relationship between spontaneous HCV clearance and sustained HIV infection control. ^41^ Co-infection reduces the possibility of the acute HCV virus clearing itself naturally. ^33^^,^^42^^,^^43^ Because HIV speeds up the development of HCV, a high viral load for this virus may also indicate a rapid progression of liver disease. ^33^^,^^37^^,^^43^ In order to take into account the additional viral load resulting from co-infection, we use the term $\varepsilon_{1}$ to impact spontaneous clearance and the term $\varepsilon_{2}$ to accelerate the disease progression, due to co-infection. ^30^ Due to the fact that HCV and HIV-1 are spread through the same ways, about 10–15 percent of acute HCV infections clear up on their own, but less than 10 percent of HIV-1 infections do. The compartmental flow diagram for the HIV-HCV co-infection model is depicted in Figure 1.

**Table 1.** List of nomenclature for HIV-HCV Model (4) are described as follows.

Variable/Parameter	Description
$S\left(t\right)$	Susceptible Individuals
$H_{U}\left(t\right)$	Unaware HIV individuals
$H_{A}\left(t\right)$	Aware HIV individuals
$H_{T}\left(t\right)$	HIV on Treatment Individuals
$A_{A}\left(t\right)$	AIDS individual
$I_{C}\left(t\right)$	Acute HCV Individual
$C_{C}\left(t\right)$	Chronic HCV Individuals
$H_{\mathrm{UI}}\left(t\right)$	Unaware HIV individual co-infected with Acute HCV
$H_{\mathrm{AI}}\left(t\right)$	Aware HIV individual co-infected with Acute HCV
$H_{\mathrm{TI}}\left(t\right)$	HIV individual on treatment co-infected with Acute HCV
$H_{\mathrm{UC}}\left(t\right)$	Unaware HIV individual co-infected with Chronic HCV
$H_{\mathrm{AI}}\left(t\right)$	Aware HIV individual co-infected with Chronic HCV
$H_{\mathrm{TI}}\left(t\right)$	HIV individual on treatment co-infected with Chronic HCV
$A_{\mathrm{AI}}\left(t\right)$	AIDS patient with acute HCV
$A_{\mathrm{AC}}\left(t\right)$	AIDS patient with chronic HCV
Λ	Recruitment rate
ω	Spontaneous clearance for Acute HCV
η	Progression rate from Acute to Chronic HCV/Non-spontaneous clearance rate
$r_{i},i=1,2,3,4,5$	HCV treatment rate for HCV
$\lambda_{H}$	Infectiousness connected to HIV infection
$\lambda_{C}$	Infectiousness connected to HIV infection
γ	Modification parameter for Acute HCV
τ	Modification parameter for Chronic HCV
$\alpha_{1},i=1,2,3$	HIV testing rate
$\delta_{i},i=1,2,3,4$	Modification parameter
$\varepsilon_{1}$	Factor that influences spontaneous HCV clearance in the presence of co-infection.
$\varepsilon_{2}$	Factor that accelerate HCV disease progression in presence of co-infection
φ	HIV infection rate among infants.
$\rho_{i},i=1,2,3$	Progression rate from unaware HIV to AIDS
$\theta_{i},i=1,2,3,4,5,6$	HIV/AIDS treatment rate
$\upsilon_{i},i=1,2,3$	HIV defaulters from treatment rate (progression rate from aware HIV to AIDs)
μ	Natural Mortality
$d_{a}$	Mortality due to AIDS
$d_{c}$	Mortality due to HCV
$\frac{1}{\sigma_{c}}$	Average time a person infected with HCV remains in an acute infection condition.
$c_{C}$	HCV contact rate
$c_{h}$	HIV contact rate
$b_{h}$	Transmission Coefficient for HIV
$b_{c}$	Transmission Coefficient for HIV

Mathematically, the flow chart leads to the system of 15 ordinary differential equations given by:

$$\begin{array}{c} \frac{\mathrm{dS}}{\mathrm{dt}}=\left(1-{\mathrm{\varphi H}}_{u}\right)\Lambda +\omega_{0}\sigma_{c}I_{c}+r_{1}C_{c}-\left(\lambda_{H}+\lambda_{C}+\mu \right)S\\ \frac{dH_{U}}{\mathrm{dt}}=\lambda_{H}S+\varphi \Lambda H_{U}+\omega \epsilon_{1}\sigma_{c}H_{\mathrm{UI}}+r_{2}H_{\mathrm{UC}}-\left(\delta_{1}\lambda_{C}+\alpha_{1}+\rho_{1}+\mu \right)H_{U}\\ \frac{dH_{A}}{\mathrm{dt}}=\alpha_{1}H_{U}+\omega \epsilon_{1}\sigma_{c}H_{\mathrm{AI}}+r_{3}H_{\mathrm{AC}}-\left(\delta_{2}\lambda_{C}+\theta_{1}+\mu \right)H_{A}\\ \frac{dH_{T}}{\mathrm{dt}}=\theta_{1}H_{A}+\omega \epsilon_{1}\sigma_{c}H_{\mathrm{TI}}+r_{4}H_{\mathrm{TC}}+\theta_{2}A_{A}-\left(\delta_{3}\lambda_{C}+\mu +\upsilon_{1}\right)H_{T}\\ \frac{dA_{A}}{\mathrm{dt}}=\rho_{1}H_{U}+\upsilon_{1}H_{T}+\omega \epsilon_{1}\sigma_{c}A_{\mathrm{AI}}+r_{5}A_{\mathrm{Ac}}-\left(\delta_{4}\lambda_{C}+\mu +d_{a}+\theta_{2}\right)A_{A}\\ \frac{dI_{C}}{\mathrm{dt}}=\lambda_{C}S-\left(\omega_{0}+\eta_{0}\right)\sigma_{c}I_{C}-\left(\gamma \;\lambda_{H}+\mu \right)I_{C}\\ \frac{dC_{c}}{\mathrm{dt}}=\eta_{0}\;\sigma_{c}I_{c}-\left(\tau \;\lambda_{H}+\mu +d_{c}+r_{1}\right)C_{c}\\ \frac{dH_{\mathrm{UI}}}{\mathrm{dt}}=\delta_{1}\lambda_{C}H_{U}+\gamma \;\lambda_{H}I_{c}-\left(\eta \;\epsilon_{2}\sigma_{c}+\alpha_{2}+\omega \epsilon_{1}\sigma_{c}+\rho_{2}+\mu \right)H_{\mathrm{UI}}\\ \frac{dH_{\mathrm{AI}}}{\mathrm{dt}}=\alpha_{2}H_{\mathrm{AI}}+\delta_{2}\lambda_{C}H_{A}-\left(\eta \;\epsilon_{2}\sigma_{c}+\theta_{3}+\omega \epsilon_{1}\sigma_{c}+\mu \right)H_{\mathrm{AI}}\\ \frac{dH_{\mathrm{TI}}}{\mathrm{dt}}=\theta_{3}H_{\mathrm{AI}}+\delta_{3}\lambda_{C}H_{T}+\theta_{4}A_{\mathrm{AI}}-\left(\eta \;\epsilon_{2}\sigma_{c}+\upsilon_{3}+\omega \epsilon_{1}\sigma_{c}+\mu \right)H_{\mathrm{TI}}\\ \frac{dH_{\mathrm{UC}}}{\mathrm{dt}}=\tau \;\lambda_{H}C_{c}+\eta \;\epsilon_{2}\sigma_{c}H_{\mathrm{UI}}-\left(r_{2}+\alpha_{3}+\rho_{3}+\mu +d_{c}\right)H_{\mathrm{UC}}\\ \frac{dH_{\mathrm{AC}}}{\mathrm{dt}}=\alpha_{3}H_{\mathrm{UC}}+\eta \;\epsilon_{2}\sigma_{c}H_{\mathrm{AI}}-\left(r_{3}+\theta_{5}+\mu +d_{c}\right)H_{\mathrm{AC}}\\ \frac{dH_{\mathrm{TC}}}{\mathrm{dt}}=\theta_{5}H_{\mathrm{AC}}+\eta \;\epsilon_{2}\sigma_{c}H_{\mathrm{TI}}+\theta_{6}A_{\mathrm{AC}}-\left(r_{4}+\upsilon_{3}+\mu +d_{c}\right)H_{\mathrm{TC}}\\ \frac{dA_{\mathrm{AI}}}{dt}=\delta_{4}\lambda_{C}A_{A}+\rho_{2}H_{\mathrm{UI}}+\upsilon_{2}H_{\mathrm{TI}}-\left(\eta \;\epsilon_{2}\sigma_{c}+\theta_{4}+\omega \epsilon_{1}\sigma_{c}+\mu +d_{a}\right)A_{\mathrm{AI}}\\ \frac{dA_{\mathrm{AC}}}{\mathrm{dt}}=\eta \;\epsilon_{2}\sigma_{c}A_{\mathrm{AI}}+\rho_{3}H_{\mathrm{UC}}+\upsilon_{3}H_{\mathrm{TC}}-\left(r_{5}+\theta_{6}+\mu +d_{a}+d_{c}\right)A_{\mathrm{AC}} \end{array}$$ (4)

### Model assumptions

• People who are being treated for HIV don't spread the virus.
• Co-infected people are approximately three times more contagious than mono-infected people. ^32^
• Persons co-infected with HIV who were not getting ART were presumed to spread HCV more easily due to higher viral loads.
• Proportional (random) mixing between all groups.
• It is assumed that an individual could be re-infected with HCV even after successful treatment if expose or engage in high-risk behaviors such as injecting drugs, ^33^ drinking alcohol, ^36^ having multiple sex partners and sex between two men ^37^ since the clearance & treatment does not confer permanent immunity. ^39^
• Treatment failure for people who have had HCV for a long time isn't taken into account because recent research has shown that newer combinations of direct-acting antivirals (DAAs) have shown cure rates of 90% to 95% in phase II and III clinical trials. ^33^
• Individuals acutely infected with HCV were assumed to spontaneously clear the virus. ^44^
• Mono-infected and co-infected people can transmit either HIV or HCV, but not both simultaneously.

Since equation (4) represents a population of humans, all of the corresponding parameters are positive. The subsequent non-negativity finding is also valid.

HIV and HCV will be analyzed independently. Thereafter, the co-infection analyses will be carried out, with positive initial conditions specified by;

$$S\left(0\right)=S_{0},H_{u}\left(0\right)=H_{U0},\;H_{A}\left(0\right)=H_{A0},\;H_{T}\left(0\right)=H_{T0},\;A_{a}\left(0\right)=A_{A0},\;I_{c}\left(0\right)=I_{C0,}\;C_{c}\left(0\right)=C_{C0},\;H_{\mathrm{uI}}\left(0\right)=H_{\mathrm{uI}0},H_{\mathrm{AI}}\left(0\right)=H_{\mathrm{AI}0},H_{\mathrm{TI}}\left(0\right)=H_{\mathrm{TI}0},A_{\mathrm{AI}}\left(0\right)=A_{\mathrm{AI}0},H_{\mathrm{UC}}\left(0\right)=H_{\mathrm{uC}0},H_{\mathrm{AC}}\left(0\right)=H_{\mathrm{AC}0},H_{\mathrm{TC}}\left(0\right)=H_{\mathrm{TC}0},A_{\mathrm{AC}}\left(0\right)=A_{\mathrm{AC}0}\in {\mathbb{R}}_{+}^{15}$$ (5)

As a result, the system dynamics ( 4) will be examined in light of the biological elements of the region

$$\Phi =\{(S\left(t\right)+H_{u}\left(t\right)+H_{A}\left(t\right)+H_{T}\left(t\right)+A_{a}\left(t\right)+I_{c}\left(t\right)+C_{c}\left(t\right)+H_{\mathrm{uI}}\left(t\right)+H_{\mathrm{AI}}\left(t\right)+H_{\mathrm{TI}}\left(t\right)+A_{\mathrm{AI}}\left(t\right)+H_{\mathrm{UC}}\left(t\right)+H_{\mathrm{AC}}\left(t\right)+H_{\mathrm{TC}}\left(t\right)+A_{\mathrm{aC}}\left(t\right))\in {\mathbb{R}}_{+}^{15}:N\leq \frac{\Lambda }{\mu }\},$$ (6)

Theorem 1:
The system variables ( 4) are positive whenever *t* > 0. In other words, Solutions of the system ( 4) with a positive initial condition will remain positive for every *t* > 0.

*Proof*:
Let $\Phi =\sup\{S\left(t\right)\geq 0,H_{u}\left(t\right)\geq 0,H_{A}\left(t\right)\geq 0,H_{T}\left(t\right)\geq 0,A_{a}\left(t\right)\geq 0,I_{c}\left(t\right)\geq 0,C_{c}\left(t\right)\geq 0,H_{\mathrm{uI}}\left(t\right)\geq 0,H_{\mathrm{AI}}\left(t\right)\geq 0,H_{\mathrm{TI}}\left(t\right)\geq 0,A_{\mathrm{AI}}\left(t\right)\geq 0,H_{\mathrm{UC}}\left(t\right)\geq 0,H_{\mathrm{AC}}\left(t\right)\geq 0,H_{\mathrm{TC}}\left(t\right)\geq 0,A_{\mathrm{aC}}\left(t\right)\geq 0.\;\text{The region}\;\Phi \in {\mathbb{R}}_{+}^{15}$ .

It follows from the model's first equation (4) that

$$\frac{\mathrm{dS}}{\mathrm{dt}}=\left(1-{\mathrm{\varphi H}}_{u}\right)\Lambda +\omega_{0}\sigma_{c}I_{c}+r_{1}C_{c}-\left(\lambda_{H}+\lambda_{C}+\mu \right)S$$

$$\frac{\mathrm{dS}}{\mathrm{dt}}=\left(1-{\mathrm{\varphi H}}_{u}\right)\Lambda +\omega_{0}\sigma_{c}I_{c}+r_{1}C_{c}\geq -\left(\lambda_{H}+\lambda_{C}+\mu \right)S$$

which is re-writeable as

$$\frac{d}{\mathrm{dt}}\left\{S,\left(t\right),e^{\left[\mathrm{\mu t}+\int_{0}^{t}\left(\lambda_{H}+\lambda_{C}\right)(\tau )d\tau \right]}\right\}\geq \Lambda \;e^{\left[\mathrm{\mu t}+\int_{0}^{t}\left(\lambda_{H}+\lambda_{C}\right)(\tau )d\tau \right]}$$

Hence,

$$S\left(\Phi \right)e^{\left[\mu \Phi +\int_{0}^{\Phi }\left(\lambda_{H}+\lambda_{C}\right)(\tau )\right]d\tau }-S\left(0\right)\geq \int_{0}^{\Phi }\Lambda \;e^{\left[\mathrm{\mu x}+\int_{0}^{x}\left(\lambda_{H}+\lambda_{C}\right)\left(\tau \right)d\tau \right]\mathrm{dx}}$$

So that

$$S\left(\Phi \right)\geq S\left(0\right)e^{\left[-\mu \Phi -\int_{0}^{\Phi }\left(\lambda_{H}+\lambda_{C}\right)(\tau )\;d\tau \right]}+\left\{e^{\left[-\mu \Phi -\int_{0}^{\Phi }-\left(\lambda_{H}+\lambda_{C}\right)(\tau )\;d\tau \right]}\right\}\int_{0}^{\Phi }\Lambda e^{\lceil \mathrm{\mu x}+\int_{0}^{x}\left(\lambda_{H}+\lambda_{C}\right)(\tau )\;d\tau \rceil \mathrm{dx}}>0$$

Analogously, it's easy to show that

$$\;H_{U}\left(t\right),H_{A}\left(t\right),H_{T}\left(t\right),A_{A}\left(t\right),I_{c}\left(t\right),C_{c}\left(t\right),H_{\mathrm{uI}}\left(t\right),H_{\mathrm{AI}}\left(t\right),H_{\mathrm{TI}}\left(t\right),H_{\mathrm{UC}}\left(t\right),H_{\mathrm{AC}}\left(t\right),H_{\mathrm{TC}}\left(t\right),A_{\mathrm{AI}}\left(t\right),\text{and}\;A_{\mathrm{AC}}\left(t\right)$$

for all t>0 are all positive.

Theorem 2:
*The closed set* $\Phi =\{\left(S\left(t\right)+H_{u}\left(t\right)+H_{A}\left(t\right)+H_{T}\left(t\right)+A_{A}\left(t\right)+I_{c}\left(t\right)+C_{c}\left(t\right)+H_{\mathrm{uI}}\left(t\right)+H_{\mathrm{AI}}\left(t\right)+H_{\mathrm{TI}}\left(t\right)+H_{\mathrm{UC}}\left(t\right)+H_{\mathrm{AC}}\left(t\right)+H_{\mathrm{TC}}\left(t\right)+A_{\mathrm{AI}}\left(t\right)+A_{\mathrm{AC}}\left(t\right)\right)\in {\mathbb{R}}_{+}^{15}:N\leq \frac{\Lambda }{\mu }\}$ is positively invariant.

*Proof:*
Now we demonstrate that every possible solution is uniformly bounded in. By adding all system ( 4) equations, we obtain:

$$N\left(t\right)=\Lambda -\mathrm{\mu N}-d_{a}\left(A_{A}+A_{\mathrm{AC}}\right)-\left(C_{c}+H_{\mathrm{UC}}+H_{\mathrm{AC}}+H_{\mathrm{TC}}+A_{\mathrm{AC}}\right)d_{a}$$ (7)

It follows from the equation that $\;\lim_{t\to \infty }\text{supN}\left(t\right)\leq \frac{\Lambda }{\mu }$ . As a result, the system's dynamics ( 4) will be looked at in light of the region’s biological factors. This is simple to demonstrate as being positively model-invariant.

Therefore as t⟶∞ , $\frac{\Lambda }{\mu }$ is the upper limit of *N* given that $\;N\left(0\right)\leq \frac{\Lambda }{\mu }$ , $N\left(t\right)\;$ will decline to this level if $\;N\left(0\right)>\frac{\Lambda }{\mu }$ . As a result, the region Φ contains all possible system solutions that can enter or remain. Under the flow caused by the system ( 4), the region of biological interest Φ is therefore positively invariant Therefore, since region Φ is positively invariant and the results for the system's existence and uniqueness hold there, it is sufficient to analyze the dynamics of the flow caused by the model ( 4) in region Φ .

## Points of equilibrium, reproduction numbers and the stability analyses

In this section, computation of disease-free equilibrium (DFE) and the endemic equilibrium (EE) will be carried out, and their stability will be examined using associative reproduction number.

## Disease-free equilibrium and the effective reproduction number

In this part, we calculate model $R_{0}^{\prime }s$ .

The effective reproduction number $R_{\mathrm{eHC}}$ , is known as the spectral radius of the next generation matrix, ^45^ governs ${E_{\mathrm{oHC}}}^{\prime }s$ linear stability. In the presence of a strategic intervention, the effective reproduction number is frequently understood as the estimated number of secondary infections produced by a single infectious individual during his/her entire infectious phase. Nevertheless, in the suggested model, the infectious persons can be classified into any of these fourteen classes $H_{U},H_{A},H_{T},A_{A},I_{C},C_{C},H_{\mathrm{UI}},H_{\mathrm{UC}},$ $H_{\mathrm{AI}},H_{\mathrm{AC}},H_{\mathrm{TI}},H_{\mathrm{TC}},$ $A_{\mathrm{AI}},A_{\mathrm{AC}}\;$ with the estimated count of secondary infections varying according to the class. Model's effective reproduction number (the total sum of secondary infections caused by HIV or HCV infected individual throughout the full contagious period in the context of treatment) is given using the latter technique as.

We will now estimate the reproduction number, $R_{\mathrm{eHC}}$ , of the entire model ( 4). Model ( 4)'s infection-free equilibrium state $E_{0\mathrm{HC}}$ is given by:

$$E_{0\mathrm{HC}}=\left[\frac{\Lambda }{\mu },0,0,0,0,0,0,0,0,0,0,0,0,0,0\right]$$

On system ( 4), we evaluate the matrices for the new transmittable terms F , the terms V , and matrix ${\mathrm{FV}}^{-1}$ , based on submission in ( 1) – ( 4) above. The reproduction number is then the spectral radius of $\;{\mathrm{FV}}^{-1}$ . $R_{0\mathrm{HC}}$ is given after some mathematical manipulation (please see the Appendix for a complete proof):

$$R_{0\mathrm{HC}}={\mathrm{FV}}^{-1}=\max\left\{R_{H},R_{C}\right\}.$$

$$R_{0\mathrm{HC}}=\max\{\frac{cb_{h}\left(1-\psi \xi \right)\left(\alpha_{1}\theta_{1}\upsilon_{1}+k_{2}k_{3}k_{4}+k_{2}k_{3}\rho_{1}-k_{2}\theta_{2}\upsilon_{1}\right)}{k_{2}\left(k_{3}k_{4}-\upsilon_{1}\theta_{2}\right)\left(\Lambda \varphi -k_{1}\right)},\frac{{\mathrm{cb}}_{c}\left(1-\psi \xi \right)\;\left(\eta_{0}\sigma_{c}-k_{5}\right)}{k_{5}\left(\eta_{0}\sigma_{c}+\omega_{0}\sigma_{c}+\mu \right)}$$ (8)

Where $k_{1}=\mu +\alpha_{1}+\rho_{1},k_{2}=\mu +\theta_{1},k_{3}=\mu +\nu_{1},k_{4}=\mu +d_{a}+\theta_{2},k_{5}=\mu +d_{c}+r_{1}$

The following lemmas is derived from Theorem 2 of Ref. 45.

Lemma 1:
If $\;R_{0\mathrm{HC}}<1$ , the disease-free equilibrium $E_{0\mathrm{HC}}$ is asymptotically stable locally, otherwise it is unstable.

By evaluating the two model sub-models listed below.

Model ( 9) is obtained from model ( 4) by equating to zero the variables pertaining to HIV dynamics $H_{U}=H_{A}=H_{T}=A_{A}=H_{\mathrm{UI}}=H_{\mathrm{UC}}=H_{\mathrm{AI}}=H_{\mathrm{AC}}=H_{\mathrm{TI}}=H_{\mathrm{TC}}=A_{\mathrm{AI}}=A_{\mathrm{AC}}=0$ , while model ( 12) is developed from model ( 4) by setting to zero the variables pertaining to HCV dynamics ( $I_{C}=C_{C}=H_{\mathrm{UI}}=H_{\mathrm{UC}}=H_{\mathrm{AI}}=H_{\mathrm{AC}}=H_{\mathrm{TI}}=H_{\mathrm{TC}}=A_{\mathrm{AI}}=A_{\mathrm{AC}}=0).$ We now compute the system's reproduction number, $R_{\mathrm{HIV}}$ ( 5). We employ the method of the next generation matrix in Ref. 45.

$$\begin{array}{c} \frac{\mathrm{dS}}{\mathrm{dt}}=\left(1-\varphi H_{U}\right)\Lambda -\left(\lambda_{H}+\mu \right)S\\ \frac{dH_{U}}{\mathrm{dt}}=\lambda_{H}S+\varphi \Lambda H_{U}-\left(k_{1}\right)H_{U}\\ \frac{dH_{A}}{\mathrm{dt}}=\alpha H_{U}-\left(k_{2}\right)H_{A}\\ \frac{dH_{T}}{\mathrm{dt}}=\theta_{1}H_{A}+\theta_{2}A_{A}-\left(k_{3}\right)H_{T}\\ \frac{dA_{A}}{\mathrm{dt}}={\upsilon H}_{T}+\rho H_{U}-\left(k_{4}\right)A_{A} \end{array}$$ (9)

Where $\lambda_{H}=c\left(1-\psi \xi \right)b_{h}\frac{H_{U}+A_{A}}{N_{h}},$ with total population given as

$$N_{h}\left(t\right)=S\left(t\right)+H_{U}\left(t\right)+H_{A}\left(t\right)+H_{T}\left(t\right)+A_{A}\left(t\right)$$

$$k_{1}=\alpha +\rho +\mu ,k_{2}=\theta_{1}+\mu ,k_{3}=\upsilon +\mu ,k_{4}=\theta_{2}+d_{a}+\mu$$

Disease-free equilibrium (DFE) evaluation of *F* and *V* generational matrices is given by

$$E_{0H}=\left[\frac{\Lambda }{\mu },0,0,0,0\right]$$ (10)

Using Ref. 22, the new infection terms matrices *F*, and the terms, *V*, are as follows:

$$F=\left(\begin{array}{cccc} c\left(1-\psi \xi \right)b_{h} & c\left(1-\psi \xi \right)b_{h} & 0 & c\left(1-\psi \xi \right)b_{h}\\ 0 & 0 & 0 & 0\\ 0 & 0 & 0 & 0\\ 0 & 0 & 0 & 0 \end{array}\right),V=\left(\begin{array}{cccc} k_{1}-\varphi \Lambda & 0 & 0 & 0\\ -\alpha & k_{2} & 0 & 0\\ 0 & -\theta_{1} & k_{3} & -\theta_{2}\\ -\rho & 0 & -\upsilon & k_{4} \end{array}\right)$$

The matrix ${\mathrm{FV}}^{-1\prime }s$ eigenvalues are as follows:

$$\left(\frac{c\left(1-\psi \xi \right)b_{h}\left(\alpha_{1}k_{3}\rho_{1}+\alpha_{1}\theta_{1}\upsilon_{1}+k_{2}k_{3}k_{4}-k_{2}\theta_{2}\upsilon_{1}\right)}{k_{2}\left(k_{3}k_{4}-\upsilon_{1}\theta_{2}\right)\left(\Lambda \varphi -k_{1}\right)},0,0,0\right)$$

The associative basic reproduction number is stated as:

$$R_{\mathrm{eH}}=\rho \left({\mathrm{FV}}^{-1}\right)=\frac{c\left(1-\psi \xi \right)b_{h}\left(\alpha_{1}k_{3}\rho_{1}+\alpha_{1}\theta_{1}\upsilon_{1}+k_{2}k_{3}k_{4}-k_{2}\theta_{2}\upsilon_{1}\right)}{k_{2}\left(k_{3}k_{4}-\upsilon_{1}\theta_{2}\right)\left(\Lambda \varphi -k_{1}\right)}$$ (11)

where ρ stands for spectral radius of ${\mathrm{FV}}^{-1}$ . The following lemma is derived from Theorem 2 of Ref. 46.

Lemma 2:
If $R_{\mathrm{eH}}<1$ , the disease-free equilibrium $E_{\mathrm{oH}}$ is asymptotically stable locally, otherwise it is unstable.

We then derive the reproduction number $R_{\mathrm{eC}}$ of model ( 12).

$$\left.\begin{array}{c} \frac{\mathrm{dS}}{\mathrm{dt}}=\Lambda +\omega \sigma_{c}I_{c}+rC_{c}-\left(\lambda_{C}+\mu \right)S\\ \frac{dI_{C}}{\mathrm{dt}}=\lambda_{C}S-\left(\omega +\eta \right)\sigma_{c}I_{C}-\mu I_{C}\\ \frac{dC_{c}}{\mathrm{dt}}=\eta \;\sigma_{c}I_{c}-\left(r+\mu +d_{c}\right)C_{c} \end{array}\right\}$$ (12)

Where $\lambda_{C}=c\left(1-\psi \xi \right)b_{c}\frac{I_{C}+C_{C}}{N_{c}},$ where $N_{c}$ is the total number of people given as

$$N_{c}\left(t\right)=S\left(t\right)+I_{C}\left(t\right)+C_{C}\left(t\right)$$ (13)

A state of HCV-free equilibrium for the system of equations in ( 12) is obtained by:

$$E_{\mathrm{oC}}=\left(S,I_{C},C_{C},\right)=\left[\frac{\Lambda }{\mu },0,0,0,0\right]$$

Using Ref. 22, the new infection terms matrices *F* and the terms *V* are thus:

$$F=\left[\begin{array}{cc} c\left(1-\psi \xi \right)b_{c} & c\left(1-\psi \xi \right)b_{c}\\ 0 & 0 \end{array}\right]$$

$$V=\left[\begin{array}{cc} \left(\omega +\eta \right)\sigma_{c}+\mu & 0\\ -\eta \sigma_{c} & \left(r+\mu +d_{c}\right) \end{array}\right]$$

The matrix ${\mathrm{FV}}^{-1\prime }s$ eigenvalues are as follows:

$$\left(\frac{c\left(1-\psi \xi \right)b_{c}\left(\eta \sigma_{c}+\mu +r+d_{c}\right)}{\left(r+\mu +d_{c}\right)\left(\mu +\left(\omega +\eta \right)\sigma_{c}\right)},0\right)$$

The associative basic reproduction number is written as:

$$R_{\mathrm{eC}}=\rho \left({\mathrm{FV}}^{-1}\right)=\frac{c\left(1-\psi \xi \right)b_{c}\left(\eta \sigma_{c}+\mu +r+d_{c}\right)}{\left(r+\mu +d_{c}\right)\left(\mu +\left(\omega +\eta \right)\sigma_{c}\right)}$$ (14)

where ρ represents the spectral radius of ${\mathrm{FV}}^{-1}$ . Therefore, the dominant eigenvalue is the basic reproduction number for HCV only model (the number of HCV infections produced by one HCV case) denoted by $\;R_{\mathrm{eC}}$ . The following lemma is derived from Theorem 2 of Ref. 45.

Lemma 3:
If $R_{\mathrm{eC}}<1$ , disease-free equilibrium $E_{\mathrm{oC}}$ is asymptotically stable locally, otherwise it is unstable.

## The endemic equilibria and stability

The following endemic equilibrium states are available in model system ( 4):

### Endemic equilibrium without HIV

From model ( 4), we set to zero variables pertaining to HIV dynamics $H_{U}=H_{A}=H_{T}=A_{A}=H_{\mathrm{UI}}=H_{\mathrm{UC}}=H_{\mathrm{AI}}=H_{\mathrm{AC}}=H_{\mathrm{TI}}=H_{\mathrm{TC}}=A_{\mathrm{AI}}=A_{\mathrm{AC}}=0$ , and is given by

$E_{C}^{*}=\left(S^{*},I_{C}^{*},C_{C}^{*},0,0,0,0,0,0,0,0,0,0,0,0\right)\;$ with

$$\left.\begin{array}{c} S^{*}=\frac{\Lambda \left(\eta \sigma_{c}+\mu +d_{c}+r\right)}{g_{1}}R_{\mathrm{eC}}\\ {I_{C}}^{*}=\frac{\left(r+\mu +d_{c}\right)c\;b_{c}\left(1-\psi \xi \right)}{g_{1}}S^{*}\\ {C_{C}}^{*}=\frac{\eta \sigma_{c}\;c\;b_{c}\left(1-\psi \xi \right)}{g_{1}}S^{*} \end{array}\right\}$$ (15)

Where $g_{1}=\left(\eta \mu \sigma_{c}+\eta d_{c}\sigma_{c}+\mu^{2}+\mathrm{\mu r}+\mu d_{c}\right)+\eta d_{c}\sigma_{c}\left(\eta \sigma_{c}+\omega \sigma_{c}+\mu \right)\left(r+\mu +d_{c}\right)$ .

Lemma 4:
The unique endemic equilibrium $E_{c}^{*}$ is said to be globally asymptotically stable for model system ( 4) if $R_{eC}>1$ and $\;R_{\mathrm{HC}}<1$ .

*Proof*:
There is no HIV in the community, so all of the HIV compartments have a value of 0. The Jacobian matrix of this three-dimensional system at endemic equilibrium $\left(S^{*},I_{C}^{*},C_{C}^{*}\right)$ , is written as

$$J\left(S^{*},I_{C}^{*},C_{C}^{*}\right)=\left[\begin{array}{ccc} \frac{cb_{c}\left(1-\psi \xi \right)\left(x_{2}+\kappa x_{3}\right)x_{1}}{{\left(x_{1}+x_{2}+x_{3}\right)}^{2}}-\frac{cb_{c}\left(1-\psi \xi \right)\left(x_{2}+\kappa x_{3}\right)}{x_{1}+x_{2}+x_{3}}-\mu & \omega \sigma_{c}-\frac{cb_{c}\left(1-\psi \xi \right)x_{1}}{x_{1}+x_{2}+x_{3}}+\frac{cb_{c}\left(1-\psi \xi \right)\left(x_{2}+\kappa x_{3}\right)x_{1}}{{\left(x_{1}+x_{2}+x_{3}\right)}^{2}} & r-\frac{cb_{c}\left(1-\psi \xi \right)\kappa x_{1}}{x_{1}+x_{2}+x_{3}}\\ -\frac{cb_{c}\left(1-\psi \xi \right)\left(x_{2}+{\mathrm{\kappa x}}_{3}\right)x_{1}}{{\left(x_{1}+x_{2}+x_{3}\right)}^{2}}+\frac{cb_{c}\left(1-\psi \xi \right)\left(x_{2}+\kappa x_{3}\right)}{x_{1}+x_{2}+x_{3}} & \frac{cb_{c}\left(1-\psi \xi \right)x_{1}}{x_{1}+x_{2}+x_{3}}-\frac{cb_{c}\left(1-\psi \xi \right)\left(x_{2}+\kappa x_{3}\right)x_{1}}{{\left(x_{1}+x_{2}+x_{3}\right)}^{2}}-\left(\omega +\eta \right)\sigma_{c}-\mu ) & \frac{cb_{c}\left(1-\psi \xi \right)\kappa x_{1}}{x_{1}+x_{2}+x_{3}}-\frac{cb_{c}\left(1-\psi \xi \right)\left(x_{2}+\kappa x_{3}\right)x_{1}}{{\left(x_{1}+x_{2}+x_{3}\right)}^{2}}\\ 0 & \eta \sigma_{c} & -r-\mu -d_{c} \end{array}\right]$$ (16)

$$\text{Trace}\;J\left(S^{*},I_{C}^{*},C_{C}^{*}\right)=-c\left(1-\psi \xi \right)b_{c}\left(I_{C}^{*}+C_{C}^{*}\right)-3\mu +c\left(1-\psi \xi \right)b_{c}S^{*}-\left(\omega_{0}+\eta_{0}\right)\sigma_{c}-d_{c}-r_{1})<0$$

$$\text{Determinant}\;J\left(S^{*},I_{C}^{*},C_{C}^{*}\right)=c\;\left(1-\psi \xi \right)b_{c}\left(\mu^{2}+\left(\eta \sigma_{c}+d_{c}+r\right)\mu +d_{c}\sigma_{c}\eta \right)I_{C}^{*}-\mu^{3}+\left(c\left(1-\psi \xi \right)\left(S^{*}-C_{C}^{*}\right)b_{c}-\left(\eta +\omega \right)\sigma_{c}-d_{c}-r\right)\mu^{2}+\left(c\left(1-\psi \xi \right)\left(\eta \sigma_{c}+d_{c}+r\right)\left(S^{*}-C_{C}^{*}\right)b_{c}-\sigma_{c}d_{c}+r\right)\left(\omega +\eta \right)\mu +\eta \sigma_{c}cb_{c}C_{C}^{*}d_{c}\left(1-\psi \xi \right)>0\;$$ (17)

As a result of $\text{trace}\left[J\right]$ being negative and the $\text{determinat}\left[J\right]$ being positive, the steady state is locally asymptotically stable.

The above statement for trace and determinant is true whenever *R*_0_ < 1 for DFE and for endemic equilibrium, whenever *R*_0_ > 1.

In order to demonstrate ${E_{C}^{*}}^{\prime }s$ global stability, firstly we observe the domain $\left\{S,I_{C},C_{C}\geq 0,S+I_{C}+C_{C}<\frac{\Lambda }{\mu }\right\}$ is positively invariant and attractive for the 3D system. Adopting Bendixson and Dulac's negative criterion to eliminate the presence of the periodic orbits using the expression $\frac{1}{I_{C}}\;\text{and}\;\frac{1}{C_{C}}$ as the Dulac multiplier, we obtain

$$\frac{S^{\prime }}{I_{C}}=\frac{\Lambda }{I_{C}}-\frac{\lambda_{c}S}{S+I_{C}+C_{C}}-\frac{\mathrm{\mu S}}{I_{C}C_{C}}+\omega +r,\frac{I_{C}^{\prime }}{I_{C}}=\frac{\lambda_{c}S}{S+I_{C}+C_{C}}-\left(\eta +\omega +\mu \right),\frac{C_{C}^{\prime }}{C_{C}}=\frac{\lambda_{c}S}{S+I_{C}+C_{C}}+\eta -\left(r+\mu +d_{c}\right)$$ (18)

When the right side of the first equation is differentiated with regards to S , the second equation with regards to $I_{c}\;$ and the right side of the third equation is differentiated with regards to $C_{c}$ ,

$$-\left(\frac{\lambda_{c}S}{S+I_{C}+C_{C}}+\frac{\lambda_{c}S}{{\left(S+I_{C}+C_{C}\right)}^{2}}\right)-\frac{\mathrm{\mu S}}{I_{C}C_{C}}<0,-\frac{\lambda_{c}S}{{\left(S+I_{C}+C_{C}\right)}^{2}}<0\;\text{and}-\frac{\lambda_{c}S}{{\left(S+I_{C}+C_{C}\right)}^{2}}<0$$ (19)

As the sum of these three expressions are negative, there is no existence of periodic orbits. Consequently, $E_{c}^{*}$ is globally asymptotic for $R_{c}>1\;\text{and}\;R_{\mathrm{HC}}<1$ .

### Endemic equilibrium without HCV

This occur by setting to zero the variables pertaining to HCV dynamics ( $I_{C}=C_{C}=H_{\mathrm{UI}}=H_{\mathrm{UC}}=H_{\mathrm{AI}}=H_{\mathrm{AC}}=H_{\mathrm{TI}}=H_{\mathrm{TC}}=A_{\mathrm{AI}}=A_{\mathrm{AC}}=0$ and is given by $S_{h}^{*},H_{U}^{*},H_{A}^{*},H_{T}^{*},A_{A}^{*},0,0,0,0,0,0,0,0,0,0$ which is present when $R_{H}>1$ exists, the endemic steady states can be computed. So that,

$$\left.\begin{array}{l} S^{*}=\frac{\Lambda \left(\Lambda \varphi -k_{1}\right)}{\Lambda \mu \varphi -\lambda k_{1}-\mu k_{1}}\\ H_{U}^{*}=\frac{\Lambda \lambda }{\Lambda \mu \varphi -\lambda k_{1}-\mu k_{1}}\\ \begin{array}{l} H_{A}^{*}=\frac{\Lambda \alpha \lambda }{\left(\Lambda \mu \varphi -\lambda k_{1}-\mu k_{1}\right)k_{2}}\\ H_{T}^{*}=\frac{\left(\alpha \theta_{1}k_{4}+\rho \theta_{2}k_{2}\right)\Lambda \lambda }{\left(k_{2}\left(\Lambda \mu \upsilon \varphi \theta_{2}-\Lambda \mu \varphi k_{3}k_{4}-\lambda \upsilon k_{1}\theta_{2}+\lambda k_{1}k_{3}k_{4}-\mu \upsilon k_{1}\theta_{2}+\mu k_{1}k_{3}k_{4}\right)\right)}\\ \;A_{A}^{*}=\frac{\Lambda \lambda \left(\alpha \upsilon \theta_{1}+\rho k_{2}k_{3}\right)}{\left(k_{2}\left(\Lambda \mu \upsilon \varphi \theta_{2}-\Lambda \mu \varphi k_{3}k_{4}-\lambda \upsilon k_{1}\theta_{2}+\lambda k_{1}k_{3}k_{4}-\mu \upsilon k_{1}\theta_{2}+\mu k_{1}k_{3}k_{4}\right)\right)} \end{array} \end{array}\right\}$$ (20)

### Endemic equilibrium of the full model

Here, the system of equations in ( 4) is set to zero and the endemic stable states are determined. $S=S^{*},H_{A}=H_{A}*,H_{T}=H_{T}*,A_{a}=A_{A}*,I_{c}={I_{c}}^{*},C_{c}={C_{c}}^{*},H_{\mathrm{uI}}=H_{\mathrm{uI}}*,H_{\mathrm{AI}}=H_{\mathrm{AI}}*,H_{\mathrm{TI}}=H_{\mathrm{TI}},\;H_{\mathrm{UC}}=H_{\mathrm{UC}}*,H_{\mathrm{AC}}=H_{\mathrm{AC}}*,H_{\mathrm{TC}}=H_{\mathrm{TC}}*A_{\mathrm{AI}}=A_{\mathrm{AI}}*\;\text{and}\;A_{\mathrm{AC}}=A_{\mathrm{AC}}*$ so that;

$$\left.\begin{array}{l} S^{*}=\frac{\left[\left(\omega_{0}+\eta_{0}\right)\sigma_{c}+\gamma \lambda_{h}+\mu \right]}{\lambda_{c}}{I_{C}}^{*}\\ {H_{U}}^{*}=\frac{\Lambda \lambda_{c}\left(\tau \lambda_{h}+k_{5}\right)+A}{\varphi \Lambda \tau \lambda_{c}\left(\tau \lambda_{h}+k_{5}\right)}{I_{C}}^{*}\\ {H_{A}}^{*}=A_{7}+A_{8}\;{I_{C}}^{*}\\ {H_{T}}^{*}=A_{24}+A_{25}\;{I_{C}}^{*}\\ {A_{A}}^{*}=A_{26}+A_{27}\;{I_{C}}^{*}\\ {I_{C}}^{*}=\frac{\Lambda \lambda_{c}\left(\tau \lambda_{h}+k_{5}\right)+A}{\lambda_{c}\left(\tau \lambda_{h}+k_{5}\right)}+\frac{\lambda_{h}\left[\left(\omega_{0}+\eta_{0}\right)\sigma_{c}+\gamma \lambda_{h}+\mu \right]}{\lambda_{c}}+\omega \;\varepsilon_{1}\sigma_{c}\left(A_{7}+\lambda_{8}\right)+r_{2}\left(A_{3}+A_{4}\right)\\ -\frac{\delta_{1}\;\left(\lambda_{c}\Lambda \left(\tau \lambda_{h}+k_{5}\right)+A\right)}{\varphi \Lambda \left(\tau \lambda_{h}+k_{5}\right)}-\frac{k_{1}\;\left(\lambda_{c}\Lambda \left(\tau \lambda_{h}+k_{5}\right)+A\right)}{\varphi \Lambda \lambda_{c}\left(\tau \lambda_{h}+k_{5}\right)}\\ {C_{C}}^{*}=\frac{\eta_{0}\sigma_{c}}{\left(\tau \lambda_{h}+k_{5}\right)}{I_{C}}^{*}\\ {H_{\mathrm{uI}}}^{*}=\frac{\delta_{1}\lambda_{c}}{k_{6}\varphi }+\frac{\left(\delta_{1}\;A+\gamma \lambda_{h}\Lambda \varphi \left(\tau \lambda_{h}+k_{5}\right)\right)}{k_{6}\varphi \Lambda \left(\tau \lambda_{h}+k_{5}\right)}{I_{C}}^{*}\\ {H_{\mathrm{AI}}}^{*}=A_{5}+A_{6}{I_{C}}^{*}\\ {H_{\mathrm{TI}}}^{*}=\frac{\theta_{3}\left(A_{5}+A_{6}A_{32}\right)}{k_{8}}+\frac{\delta_{3}\lambda_{c}\left(A_{24}+A_{25}A_{32}\right)}{k_{8}}+\frac{\theta_{4}\left(\frac{\eta \epsilon_{2}\sigma_{c}\delta_{1}\lambda_{c}}{k_{6}k_{9}\varphi }+A_{1}A_{32}\right)}{k_{8}}\\ {H_{\mathrm{uC}}}^{*}=\frac{\eta \;\varepsilon_{2}\sigma_{c}\delta_{1}\lambda_{c}}{k_{6}k_{9}\varphi }+A_{1}{I_{C}}^{*}\\ {H_{\mathrm{AC}}}^{*}=A_{3}+A_{4}{I_{C}}^{*}\\ {H_{\mathrm{TC}}}^{*}=A_{9}+A_{10}{I_{C}}^{*}\\ {A_{\mathrm{AI}}}^{*}=A_{30}+A_{31}{I_{C}}^{*}\\ {A_{\mathrm{AC}}}^{*}=A_{28}+A_{29}{I_{C}}^{*} \end{array}\right\}$$ (21)

Where the values of $k_{1}-k_{13}\;\text{and}\;A_{1}-A_{31}$ are given in appendix A, available from the source code link provided.

We want to consider how the reproduction number of HCV, $R_{C}$ and reproduction number of HIV $R_{H}$ impact one another as follows:

$$\frac{\partial R_{H}}{\partial C}*\frac{\partial C}{\partial R_{C}}=\frac{\partial R_{H}}{\partial R_{C}}=\frac{b_{h}Mk_{5}\left(\eta_{0}\sigma_{c}+\omega_{0}\sigma_{c}+\mu \right)}{k_{2}\left(\Lambda \varphi -k_{1}\right)\left(\theta_{2}\mu +\left(\mu +\nu_{1}\right)\left(\mu +d_{a}\right)\right)b_{c}\left(\eta_{0}\sigma_{c}-k_{5}\right)}\;$$ (22)

which is the total sum of new HIV infections that one person with HIV will cause in a population where HCV is already common. Even if $R_{C}>1>R_{H}$ , HIV will be allowed to spread into a population where HCV is common if $R_{\mathrm{HC}}$ is greater than 1. In other words, $R_{\mathrm{HC}}>1$ which shows the presence of HCV makes it easier for HIV to spread in a community. But for $\;R_{\mathrm{HC}}<1$ , HCV is still the biggest health issue, even though HIV has been spread to a population where HCV was already common and vice versa.

Taking the partial derivative of $R_{\mathrm{HC}}$ in ( 22) with regards to $\;b_{h}$ , we have

$$\frac{\partial R_{\mathrm{HC}}}{\partial b_{h}}=\frac{c_{h}Mk_{5}\left(\eta_{0}\sigma_{c}+\omega_{0}\sigma_{c}+\mu \right)\;}{k_{2}\left(\Lambda \varphi -k_{1}\right)\left(\theta_{2}\mu +\left(\mu +\nu_{1}\right)\left(\mu +d_{a}\right)\right)c_{c}b_{c}\left(\eta_{0}\sigma_{c}-k_{5}\right)}>0.$$ (23)

Where $M=\left(\alpha_{1}\theta_{1}\upsilon_{1}+k_{2}k_{3}\rho_{1}+k_{2}\left(\mu +\nu_{1}\right)\left(\mu +d_{a}\right)+k_{2}\theta_{2}\mu \right)$

Any time $R_{c}>1$ , equation (14)'s positive result shows that the existences of HCV accelerates the spread of HIV infections in a community and vice versa.

From ( 22) since the partial derivatives with respect to $R_{C}$ is positve, this signifies that as the reproduction number of HCV, $R_{C}$ increases, it impacts the reproduction number of HIV $\;R_{H}$ . Then, we should simply allow HCV infection to reduce to avoid increased viral load in HIV-infected individuals because any slight increase in HCV will make HIV increase.

Furthermore, from ( 23) since the partial derivatives with respect to *b*_*h*_ is also positive, this simply implies that parameter *b*_*h*_ is sensitive to reproduction number of HIV-HCV. The implication is that any increase in transmission coefficient of HIV; *b*_*h*_, will result in increase in the reproduction number of HIV-HCV. In the same manner, any increase in transmission coefficient of HCV; *b*_*c*_, will result in increase in the reproduction number of HIV-HCV. This also suggest that these parameters are parameters to watch out for when controlling the transmission of HIV and HCV coinfection.

## The global stability of the disease free equilibria

Computation of global stability of the disease-free equilibrium of the whole model ( 4) is done in this section. To start, we will calculate the stability of the disease-free equilibria of both of the sub-models ( 9) and ( 12).

Theorem 3:
Disease-free equilibrium $E_{0H}\;$ is globally asymptotically stable for model ( 9) if $R_{0H}$ is less than 1.

*Proof:*
Here, the Comparison theorem as outlined by Refs. 47– 49 is applied. The rate of change of the system's infected components ( 9) can be expressed as:

$$\left(\begin{array}{c} \frac{dH_{U}}{\mathrm{dt}}\\ \frac{dH_{A}}{\mathrm{dt}}\\ \frac{dH_{T}}{\mathrm{dt}}\\ \frac{dA_{A}}{\mathrm{dt}} \end{array}\right)=\left(F-V\right)\left(\begin{array}{c} H_{U}\\ H_{A}\\ H_{T}\\ A_{A} \end{array}\right)-\left(1-\frac{S_{h}}{N_{h}}\right)F\left(\begin{array}{c} H_{U}\\ H_{A}\\ H_{T}\\ A_{A} \end{array}\right)$$

Since the disease-free $H_{U}=H_{A}=H_{T}=A_{A}=0\to \left(0,0,0,0\right)$ and $S_{h}\leq N_{h}$ , as t→∞ in $\;\Gamma_{h}$ , thus,

$$\begin{array}{c} \left(\begin{array}{c} \frac{dH_{U}}{\mathrm{dt}}\\ \frac{dH_{A}}{\mathrm{dt}}\\ \frac{dH_{T}}{\mathrm{dt}}\\ \frac{dA_{A}}{\mathrm{dt}} \end{array}\right)\leq \left(F-V\right)\left(\begin{array}{c} H_{U}\\ H_{A}\\ H_{T}\\ A_{A} \end{array}\right)\left(\begin{array}{c} \frac{dH_{U}}{\mathrm{dt}}\\ \frac{dH_{A}}{\mathrm{dt}}\\ \frac{dH_{T}}{\mathrm{dt}}\\ \frac{dA_{A}}{\mathrm{dt}} \end{array}\right)\leq \left(\left(\begin{array}{c} \left(c\left(1-\psi \xi \right)b_{h}+\Lambda \varphi -k_{1}\right)\;\\ \alpha \\ 0\\ \rho \end{array}\begin{array}{c} c\left(1-\psi \xi \right)b_{h}\\ -k_{2}\\ \theta_{1}\\ 0 \end{array}\begin{array}{c} 0\\ 0\\ -k_{3}\\ \upsilon \end{array}\begin{array}{c} c\left(1-\psi \xi \right)b_{h}\\ 0\\ \theta_{2}\\ -k_{4} \end{array}\right)\right)\left(\begin{array}{c} H_{U}\\ H_{A}\\ H_{T}\\ A_{A} \end{array}\right) \end{array}$$

If $R_{0H}<1$ , then $\rho \left(F-V\right)<1$ , which is the same as stating that all eigenvalues of the matrix F−V lie in the left-half plane. ^45^ Therefore, the linear system described by the equality (23) is stable anytime $R_{0H}<1\;$ and $H_{U}=H_{A}=H_{T}=A_{A}=0\to \left(0,0,0,0\right)\;\mathrm{as}\;t\to \infty$ for this linear ordinary differential equation (ODE) system. As a result of employing a basic comparison theorem, ^48^^–^^50^ we obtain $H_{U}=H_{A}=H_{T}=A_{A}=0\to \left(0,0,0,0\right)\;$ for the nonlinear system ( 9) represented by the last four equations of the system. We construct a linear system with $S\left(t\right)=\frac{\Lambda }{\mu }$ by inserting $H_{U}=H_{A}=H_{T}=A_{A}=0$ into the first equation of model ( 9). Thus, $\left(S\left(t\right),H_{U},H_{A},H_{T},A_{A}\right)\to \left(\frac{\Lambda }{\mu },0,0,0,0\right)\mathrm{as}\;t\to \infty$ for $\;R_{0H}<1$ , so $E_{0H}\;$ is asymptotically stable globally if $R_{0H}<1$ .

Now, we follow the same approach to compute the global stability of the disease-free equilibrium of the sub model ( 9).

Theorem 4:
If $\;R_{0C}<1$ the disease-free equilibrium $E_{0C}\;$ in submodel ( 9) is globally asymptotically stable.

*Proof*:
Here, the Comparison theorem as outline by Refs. 48, 49 is applied. The rate of change of the system's acute and chronic components (8) can be expressed as:

$$\left(\begin{array}{c} \frac{dI_{C}}{\mathrm{dt}}\\ \frac{dC_{C}}{\mathrm{dt}} \end{array}\right)=\left(F-V\right)\left(\begin{array}{c} I_{C}\\ C_{C} \end{array}\right)-\left(1-\frac{S_{c}}{N_{c}}\right)F\left(\begin{array}{c} I_{C}\\ C_{C} \end{array}\right)$$

$I_{C}=C_{C}=0\to \left(0,0\right)$ and $S_{c}\leq N_{c}$ , as t→∞ in $\Gamma_{\mathrm{cv}}$ . Thus,

$$\left(\begin{array}{c} \frac{dI_{C}}{\mathrm{dt}}\\ \frac{dC_{C}}{\mathrm{dt}} \end{array}\right)\leq \left(F-V\right)\left(\begin{array}{c} I_{C}\\ C_{C} \end{array}\right)$$

$$\left(\begin{array}{c} \frac{dI_{C}}{\mathrm{dt}}\\ \frac{dC_{C}}{\mathrm{dt}} \end{array}\right)\leq \left(\left(\begin{array}{c} \left(c\left(1-\psi \xi \right)b_{c}-\left(\omega +\eta \right)\sigma_{c}-\mu -\lambda \right)\;\\ -\eta \sigma_{c} \end{array}\begin{array}{c} c\left(1-\psi \xi \right)b_{c}\kappa \\ r+\mu +d_{c}-\lambda \end{array}\right)\right)\left(\begin{array}{c} I_{C}\\ C_{C} \end{array}\right)$$ (24)

If $\;R_{0C}<1$ , then $\rho \left(F-V\right)<1$ , which is the same as stating that all eigenvalues of the matrix F−V lie in the left-half plane. Therefore, the linear system described by the equality ( 12) anytime $R_{0C}<1\;$ and $I_{C}=C_{C}=0\to \left(0,0\right)\;\mathrm{as}\;t\to \infty$ for this linear ordinary differential equation (ODE) system. As a result of employing a basic comparison theorem, ^48^^,^^50^ we derived $I_{C}=C_{C}=0\to \left(0,0\right)\;$ for the nonlinear system ( 12) represented by the last two equations of the system. We construct a linear system with $S\left(t\right)=\frac{\Lambda }{\mu }$ by inserting $I_{C}=C_{C}=0\;$ into the first equation of model ( 12). Thus, $\left(S\left(t\right),I_{C},C_{C}\right)\to \left(\frac{\Lambda }{\mu },0,0\right)\mathrm{as}\;t\to \infty$ for $\;R_{0C}<1$ , so $E_{0C}\;$ is asymptotically stable globally if $\;R_{0C}<1$ .

Model ( 4)'s disease-free equilibrium can only be globally stable under very narrow circumstances, namely when new co-infection cases are avoided. In such circumstances, patients with HIV or HCV infections could not get both diseases.

Theorem 5:
The global asymptotically stable HIV-HCV disease-free equilibrium $E_{0}$ ( 4) is unstable if $R_{\mathrm{HC}}>1$ and stable if $R_{\mathrm{HC}}<1$ .

*Proof*:
The Refs. 48, 49 Comparison approach is employed here.

Check appendix B for the proof of the GSA of the full model available from the source code link provided.

## Numerical simulation

In this part, we use the Maple computer language to perform in-depth numerical simulations to assess the effects of HCV treatment and antiretroviral therapy in dual-infected populations under various beginning conditions. Table 2 lists the parameter values we utilize for our numerical simulations.

**Table 2.** Parameters used in the numerical simulations of model.

Parameters	Parameters value	Source
Λ	29 yr ^−1^	^19^
φ	0.02	[Assumed]
$c_{h}$	3 patners/yr	^51^
$c_{c}$	2 patners/yr	Assumed
$b_{h}$	0.036	^22^
$b_{c}$	0.05	^22^
μ	0.02 0	^22^
$\alpha_{1},i=1,2,3$	0.65	[Assumed]
$\rho_{i},i=1,2,3$	0.322	[Assumed]
$\upsilon_{i,}i=1,2,3$	0.0169	^52^
$\theta_{1}$	1.6949	Assumed
$\theta_{1},i=1,2,3,4,5$	1.6949	^53^
$d_{a}$	0.333 yr ^−1^	^51^
$d_{c}$	0.005	^40^
ψξ	0.02	^54^
$1/\sigma_{c}$	5.8 months	^29^
η	0.43	^29^
$r_{i},i=1,2,3,4,5$	3.3	^55^
ω	0.25	^56^
$\varepsilon_{1}$	2.23	^29^
$\varepsilon_{2}$	1.15	^29^
$\kappa_{i},i=1,2$	1.0002	[Assumed]

**Figure 2.**
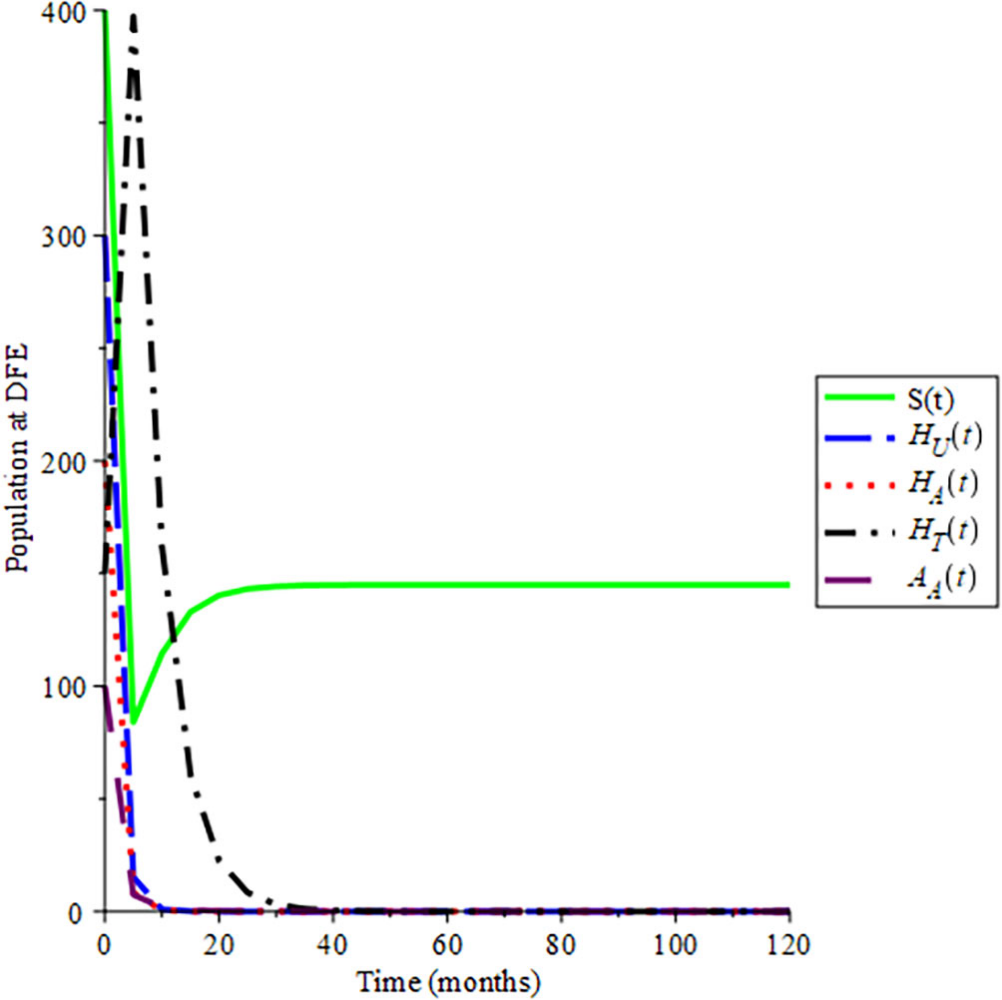
Proportion of different population of HIV at DFE when *R*_0_ < 1.

**Figure 3.**
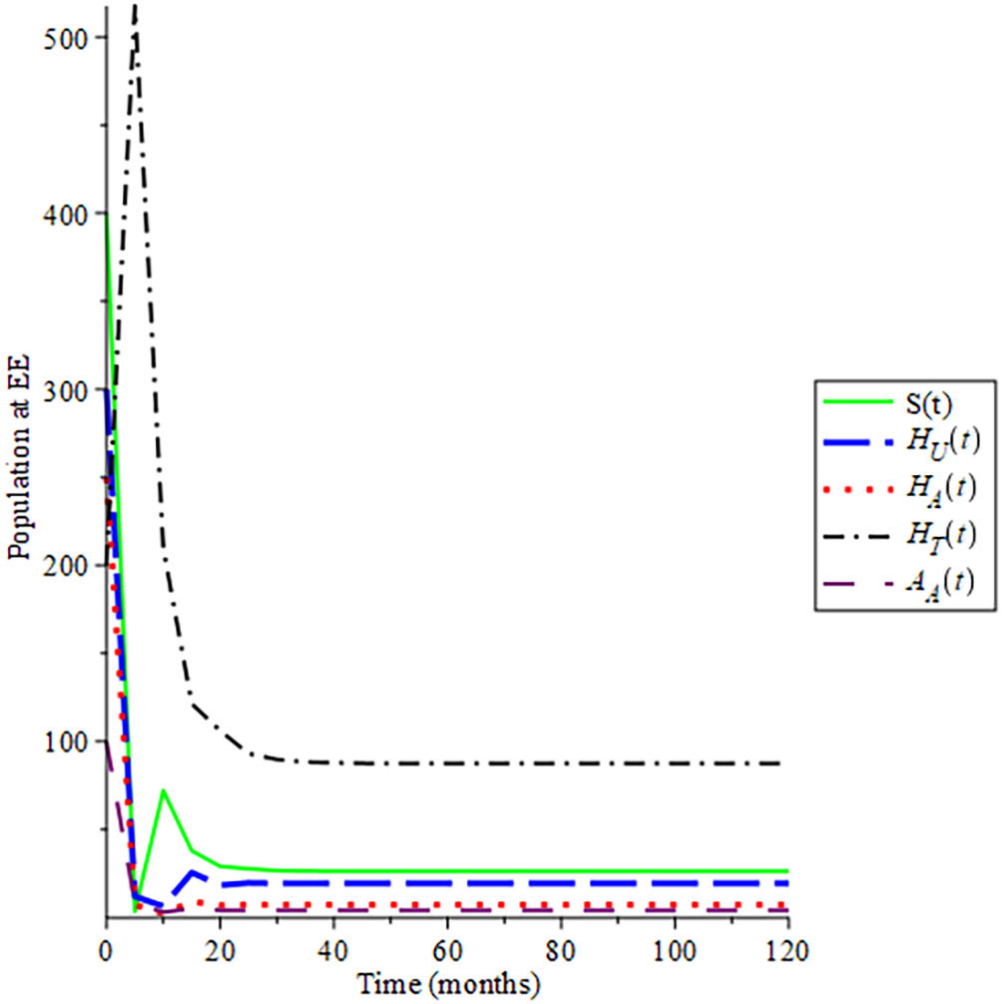
Proportion of different population HIV at EE when *R*_0_ > 1.

Selecting 100 different initial conditions, Figure 2 show that the trajectories of the solutions converge to (145, 0, 0, 0, 0), Hence, $R_{\mathrm{eH}}=0.712$ , this aids the result in Theorem 3 that the disease-free equilibrium is globally asymptotically stable if $R_{\mathrm{eH}}<1$ . Also, the endemic equilibrium trajectories of the solutions converge to (8.420; 22.353; 17.485; 91.452; 4.534) in Figure 3 choosing different initial conditions, for a given parameter values and initial conditions given in Table 2 respectively, hence $R_{\mathrm{eH}}=7.1234$ . This again supports Lemma 4 that the endemic equilibrium is globally asymptotically stable if $\;R_{\mathrm{eH}}>1$ .

**Figure 4.**
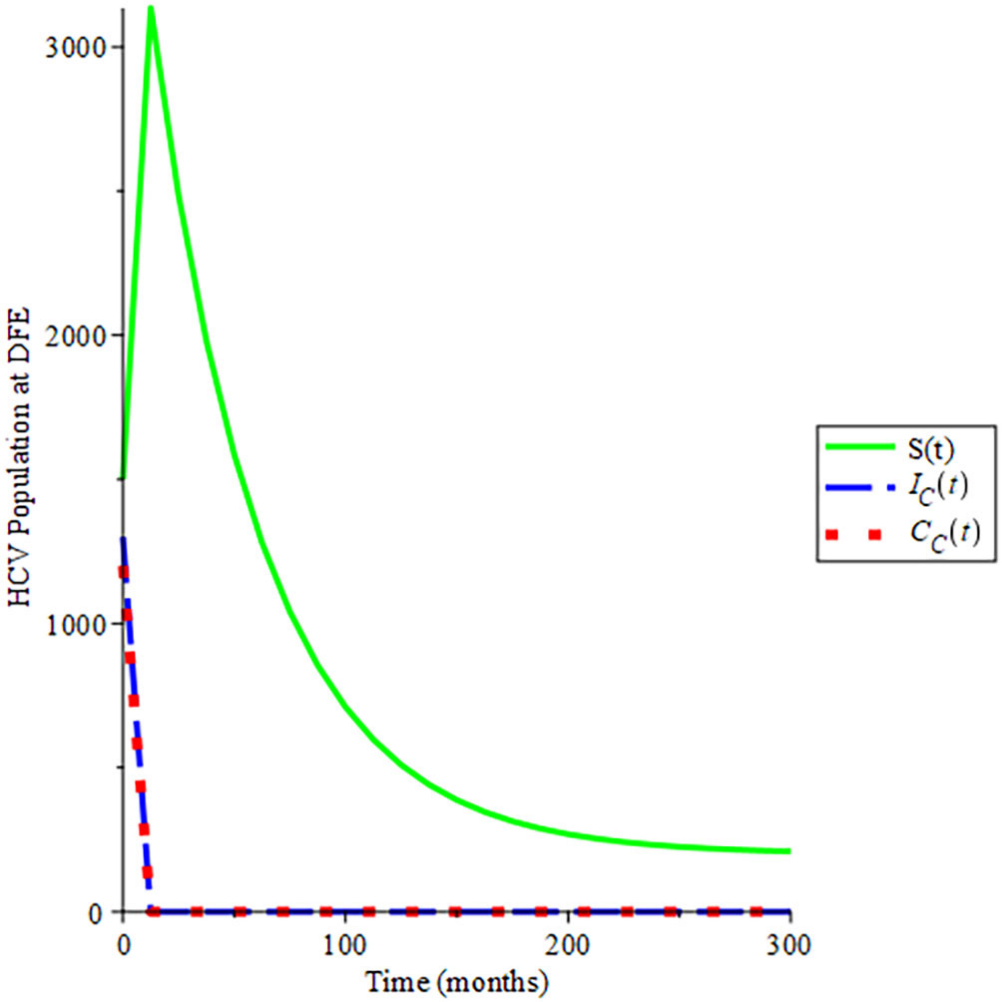
Proportion of different population of HCV at DFE when *R*_0_ < 1.

Figure 4, shows the behavioural dynamics of the HCV populations when $\;R_{\mathrm{ec}}<1.$ Over time, a gradual increase in the susceptible population is obtained which later remains stable and does not tend to zero while acute HCV and chronic HCV tend to zero when $R_{\mathrm{ec}}\;$ is less than unity. This is an indication that the susceptible population will never be zero and endemicity will not exist. As such the disease will die over time due to the basic reproduction number of less than one, and the trajectories of the solution converge to $\left(200,0,0\right),$ hence $R_{\mathrm{ec}}=0.1010$ which authenticates the analysis shown in Theorem 4, that the disease-free equilibrium is globally asymptomatically stable if $R_{\mathrm{ec}}<1$ . This indicates that disease dies out early which is influenced by effective condom use and other strategies.

**Figure 5.**
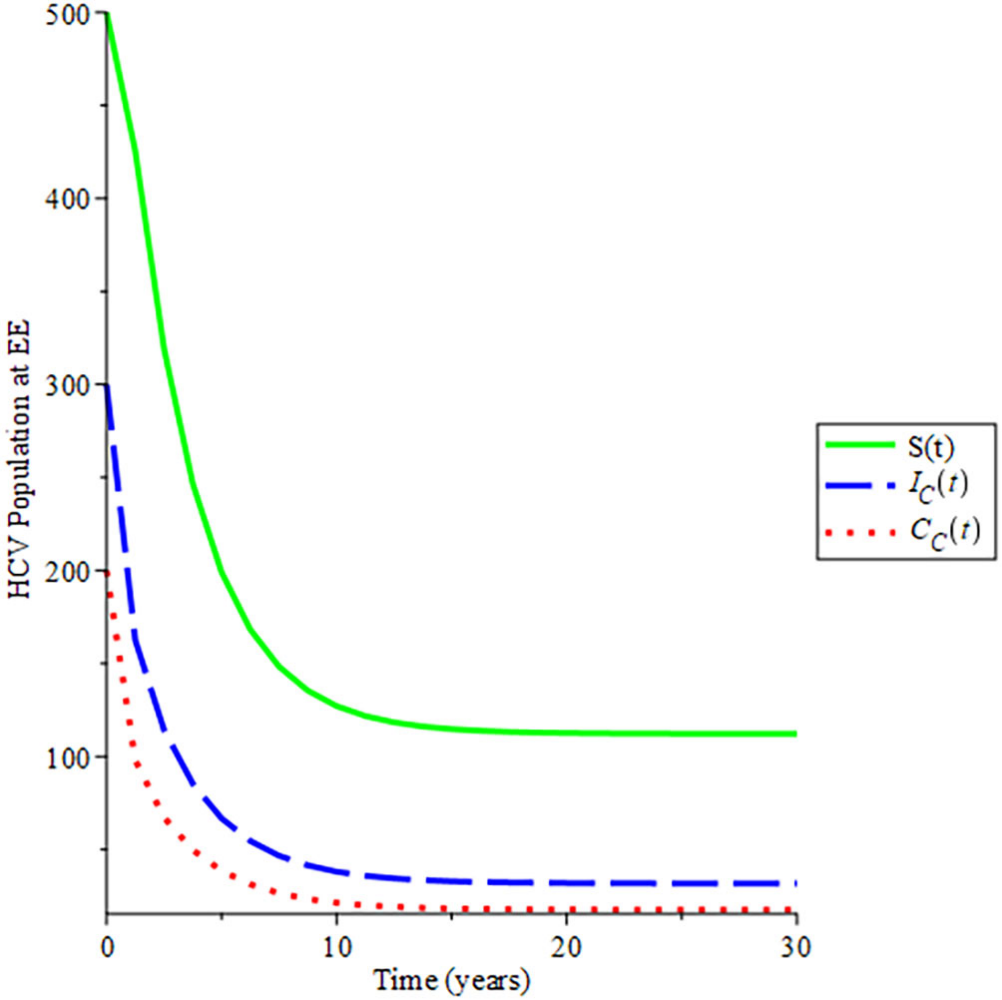
Proportion of different population of HCV at EE when *R*_0_ > 1.

The behavioural dynamics of the susceptible, acute HCV and chronic HCV populations in endemic states was shown in Figure 5. Each system approached asymptotically the stable HCV endemic equilibrium state of system 12. Moreover, the endemic equilibrium trajectories of the solution converge to $\left(234.034,120.894,89.469\right)\;$ by choosing different initial conditions for given parameters in Table 2, hence, $\;R_{\mathrm{ec}}=1.011.$ This again aids Theorem 3.12 that the endemic equilibrium is globally asymptomatically stable if $R_{\mathrm{ec}}>1$ .

**Figure 6.**
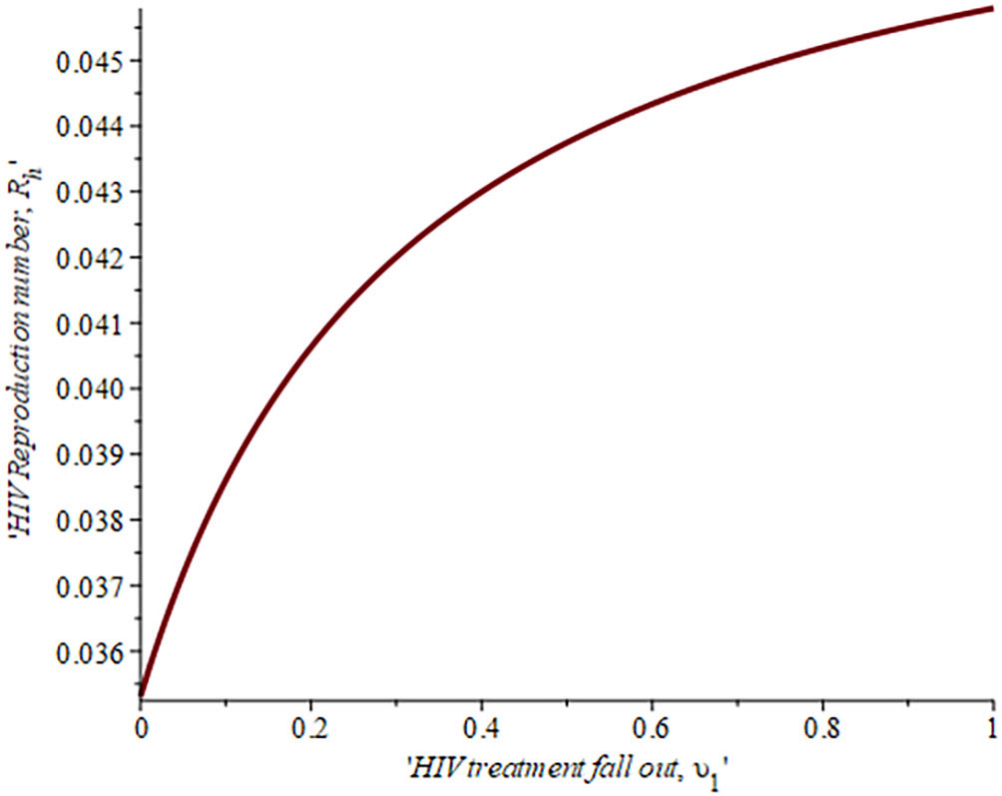
Impact of HIV treatment fall-out population on HIV reproduction number.

**Figure 7.**
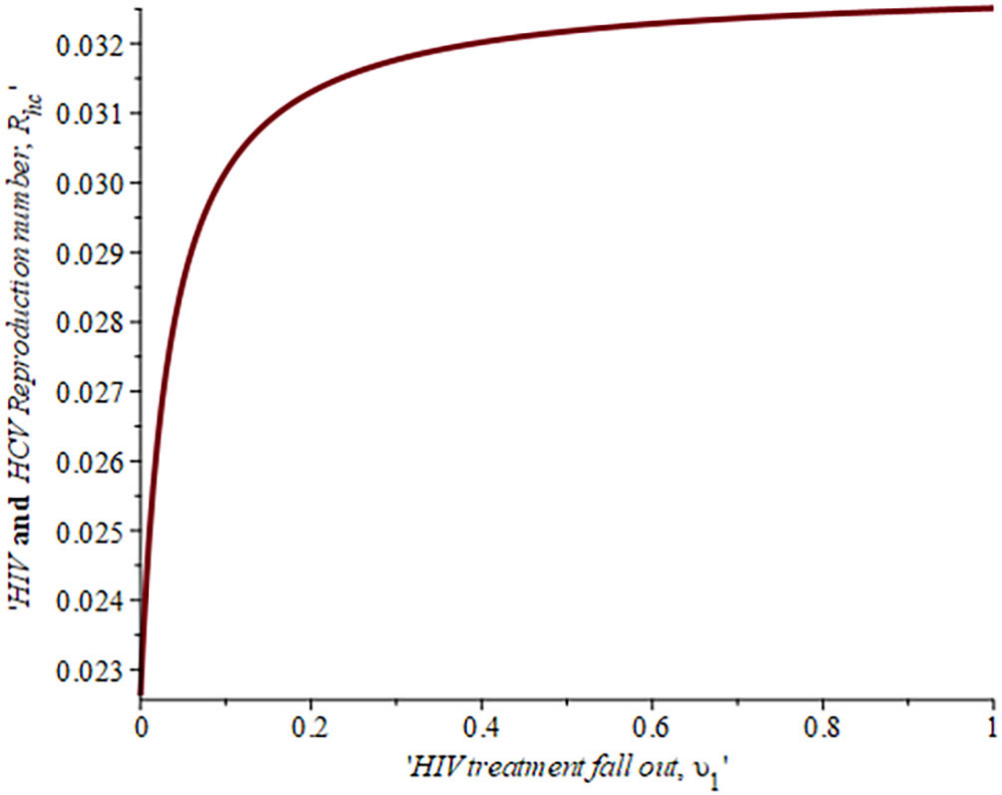
Impact of HIV treatment fall-out population on HIV-HCV co-infected reproduction number.

Figure 6, shows the impact of fall-out on the HIV reproduction number, $R_{\mathrm{eH}}$ . As the proportion of the fallout population increases HIV reproduction also increases. For example, if the proportion of the population that fall-out of treatment is $16.4\%,\;R_{\mathrm{eH}}=0.04,\;\text{if}\;\upsilon =30\%,\;R_{\mathrm{eH}}=0.042\;\text{and when}\;\upsilon =50\%\;R_{\mathrm{eH}}=0.044$ , this supports the data fitting done by Ref. 52. Figure 7 shows the impact of fall-out on the dually infected with HIV-HCV reproduction number. As the proportion of the fallout population increases HIV reproduction also increases. For example, if the proportion of the population that fall-out of treatment is $16.4\%,\;R_{\mathrm{eH}}=0.04,\;\text{if}\;\upsilon =30\%,\;R_{\mathrm{eH}}=0.042\;\text{and when}\;\upsilon =50\%\;R_{\mathrm{eH}}=0.044$ , this supports the data fitting done by Ref. 52.

**Figure 8.**
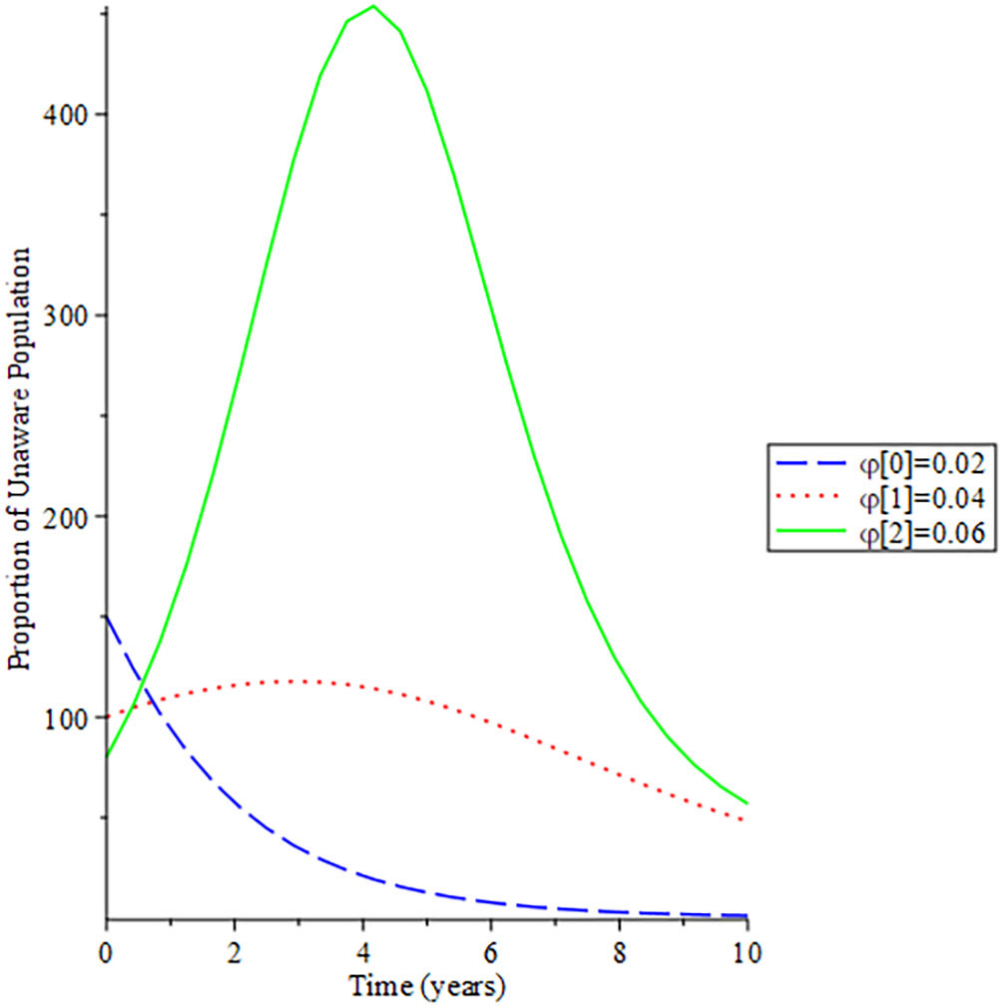
Behavioral dynamics of infected HIV unaware when varying vertical transmission φ with time.

**Figure 9.**
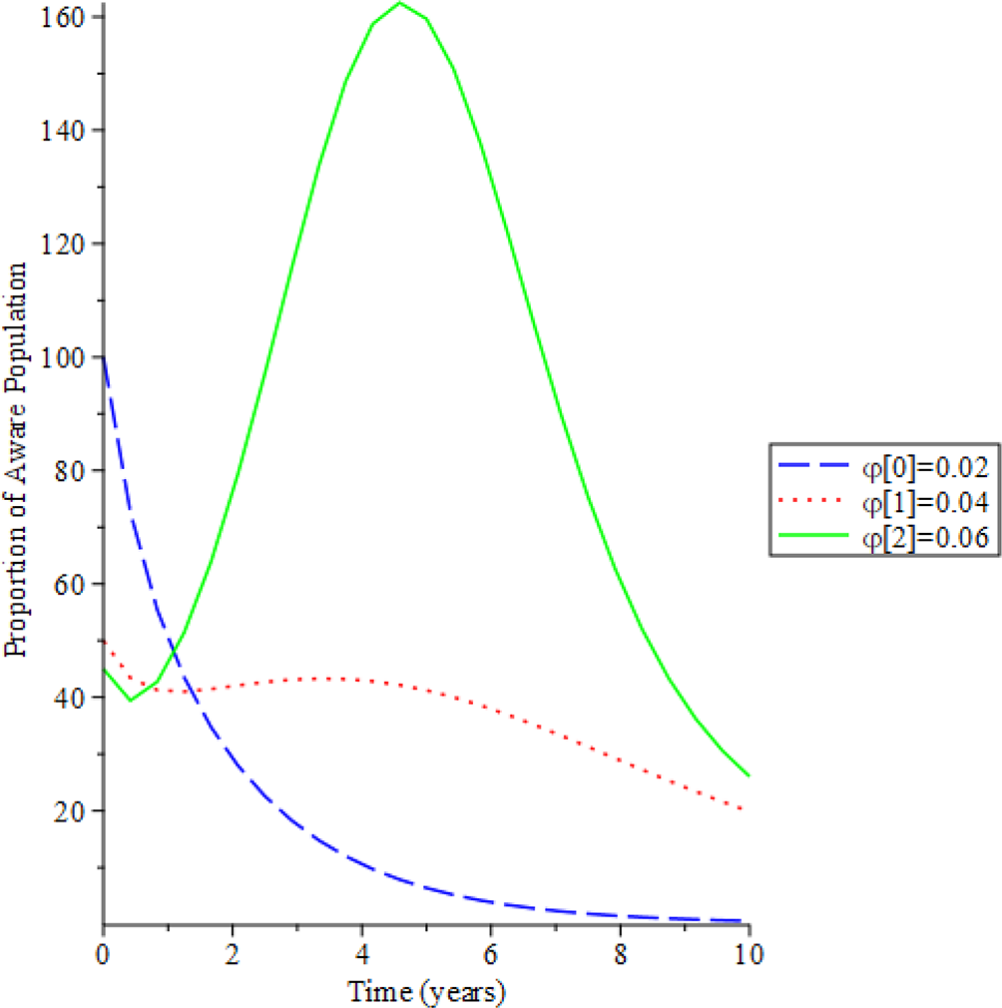
Behavioral dynamics of Infected HIV awareness when varying vertical transmission φ with time.

**Figure 10.**
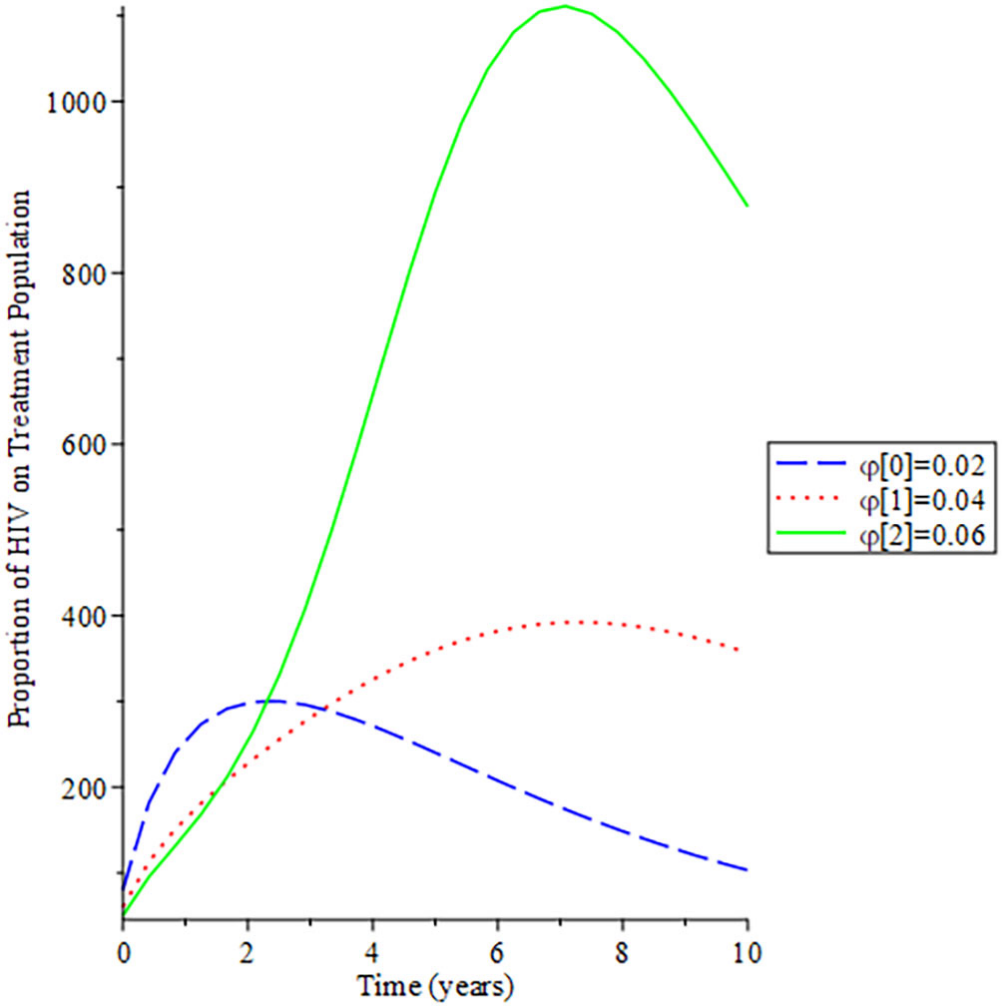
Behavioral dynamics of Infected HIV on treatment population when varying vertical transmission φ with time.

**Figure 11.**
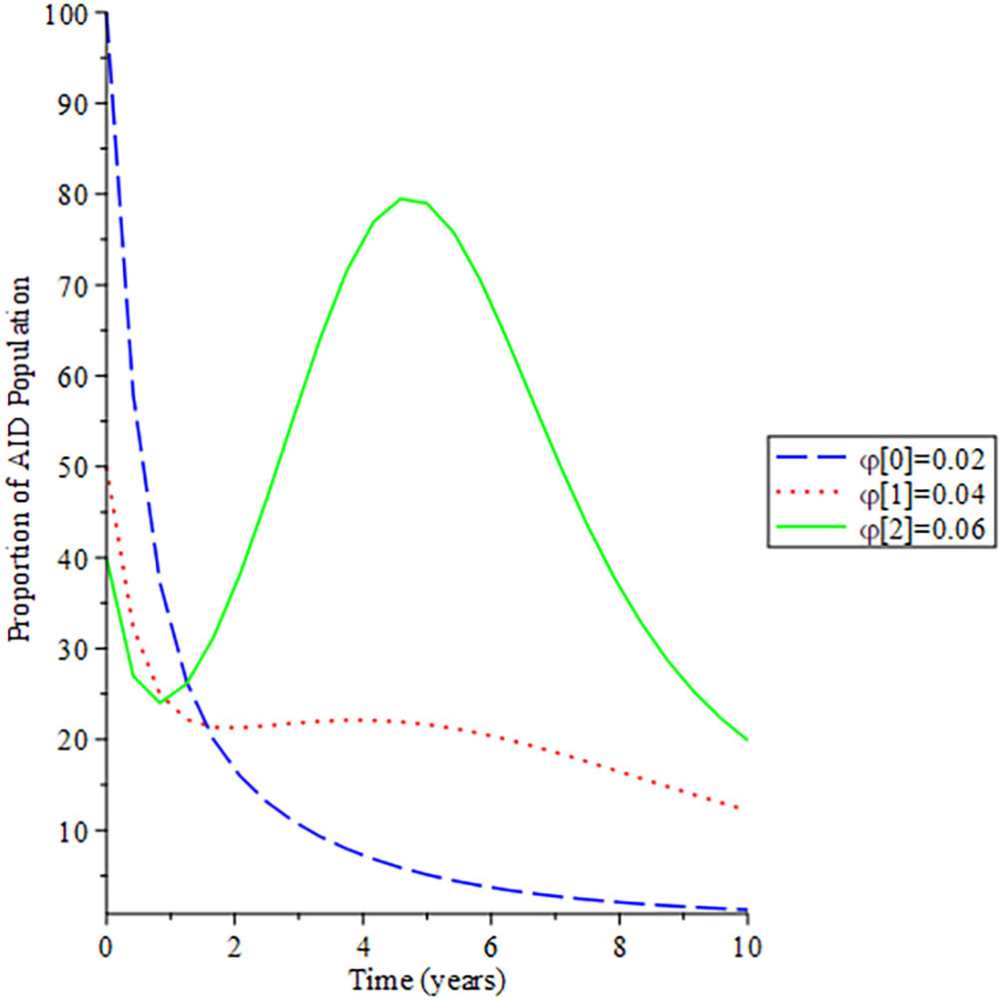
Behavioral dynamics of AIDS population when varying vertical transmission φ with time.

**Figure 12.**
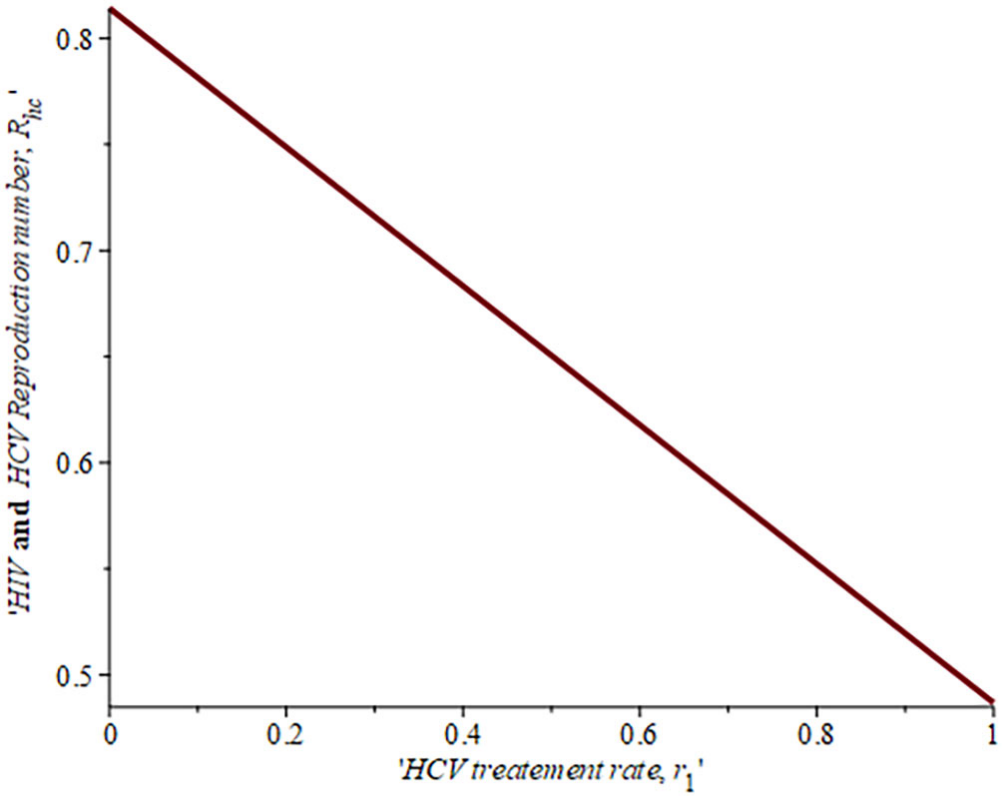
Impact of HCV treatment rate on HIV-HCV co-infection reproduction number.

**Figure 13.**
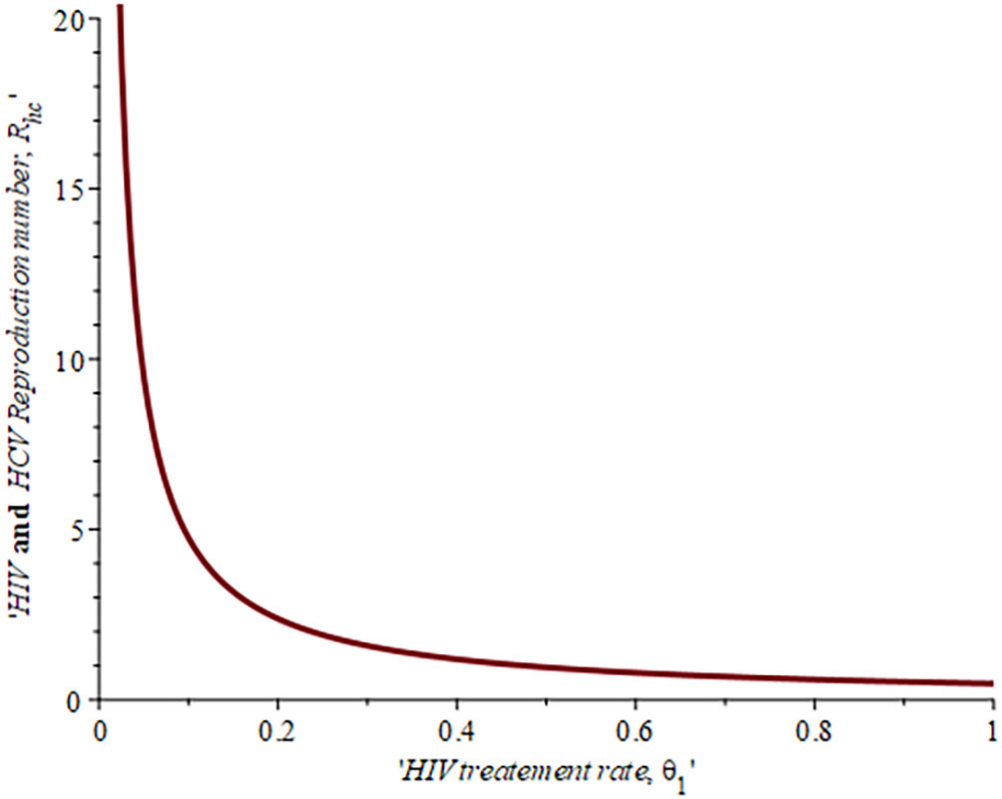
Impact of HIV treatment rate on HIV-HCV co-infection reproduction number.

Figures 8- 11 show the impact of vertical transmission on the dynamics of the HIV/AIDS infected classes. From these figures, even with a 2% increment in the population, there is a significant increase in the dynamics of the infected class. Figure 12 gives the impact of treating HCV first on the HIV-HCV co-infection population. The linear contour plot shows that when (0.60) 60% of the co-infected individual is treated for HCV the reproduction number $R_{\mathrm{ech}}\;\text{is}\;0.61\;\left(61\%\right)$ , also if we treat (0.80) 80% of the individual who are co-infected of their HCV first the $\;R_{\mathrm{ech}}\;$ reduces to 0.55 (55%). The plot depicts that if we treat more of the dually infected population with HCV first, the transmission rate of the co-infection will be reduced by 0.14% thereby lowering the danger of liver cancer and death due to HIV/AIDS or death due to HCV. Likewise, Figure 13 depict the impact of treating HIV first on the HIV-HCV co-infection population. The linear contour plot shows that when (0.60) 60% of the co-infected individual is treated for HIV the reproduction number $R_{\mathrm{ehc}}\;\text{is}\;0.906\;\left(90.6\%\right)$ , also if we treat (0.8) 80% of the individual who are co-infected of their HIV first the $\;R_{\mathrm{ehc}}\;$ reduces to 0.725 (72.5%). The plots depicts that treating more of the dually infected population with HCV first, the transmission rate of the co-infection more than treating HIV first in co-infected patient, which thereby lowering the danger of liver cancer and death due to HIV/AIDS or death due to HCV.

**Figure 14.**
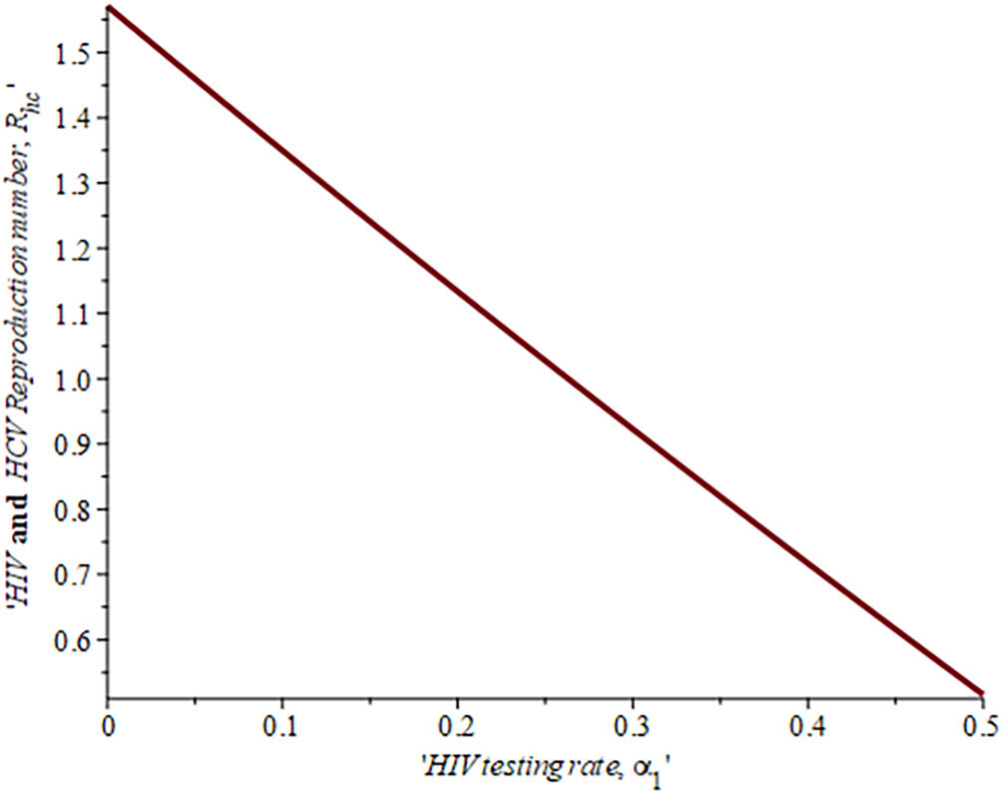
Impact of HIV testing rate on HIV-HCV co-infection reproduction number.

**Figure 15.**
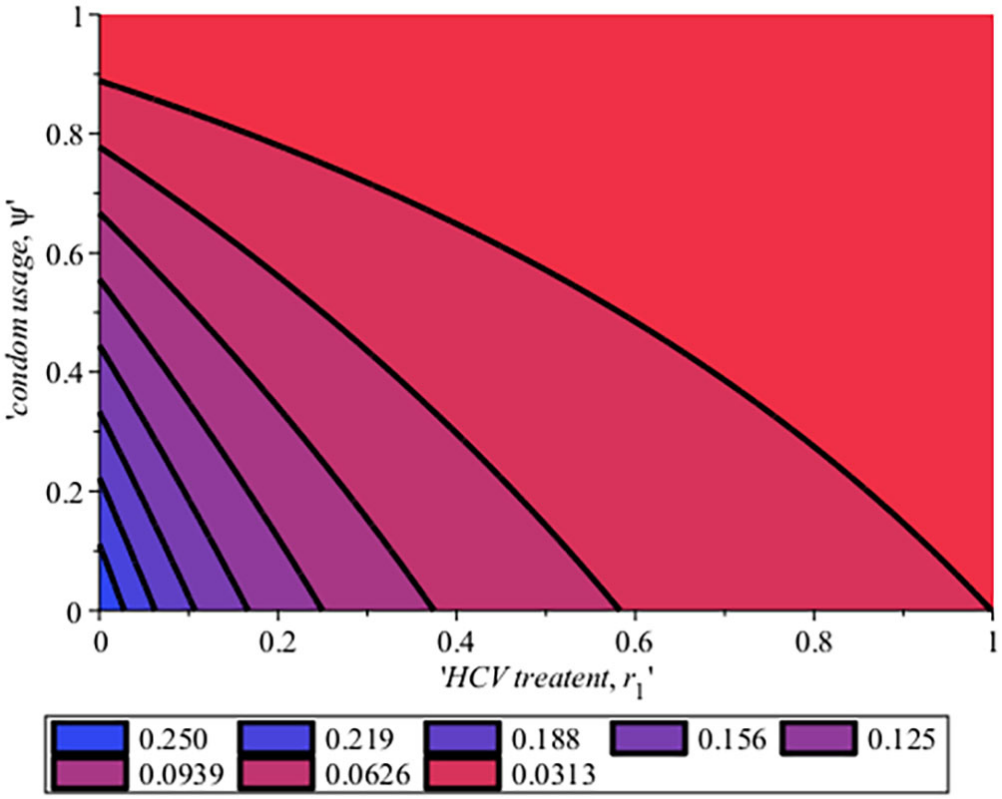
The effect of treatment and condom use on HCV reproduction number for HCV.

**Figure 16.**
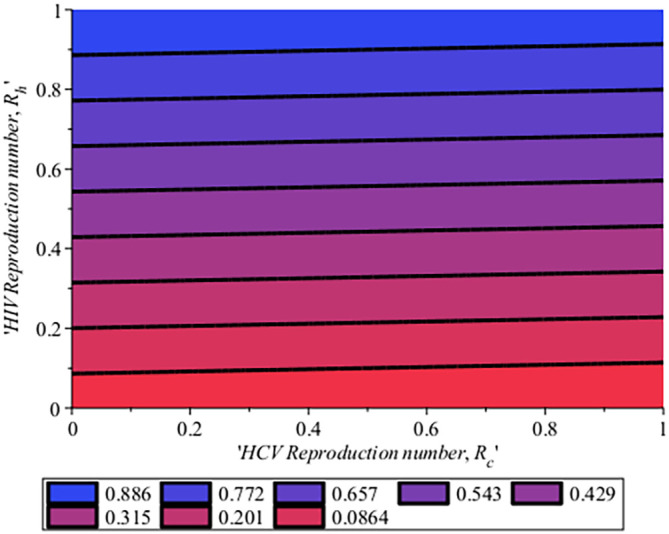
Impact of HCV reproduction number on HIV reproduction number.

**Figure 17.**
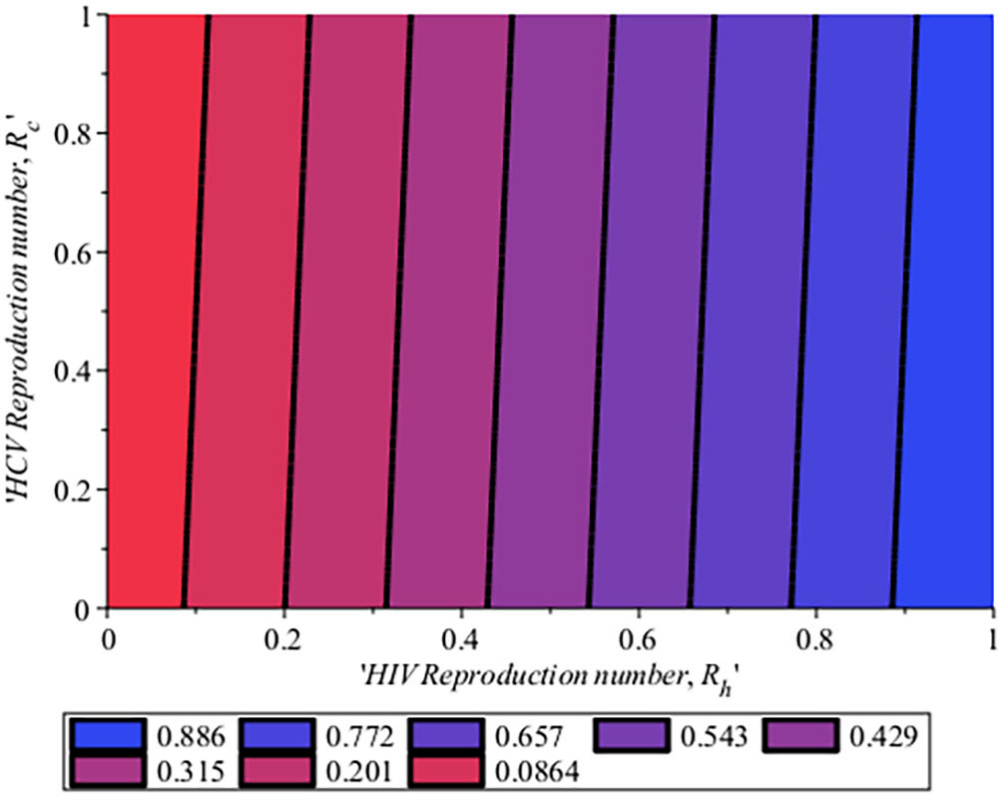
Impact of HIV reproduction number on HCV reproduction number.

Figure 14 described the impact of testing on the HIV-HCV co-infection population. The plot shows that when (0.30) 30% of the co-infected individual is tested for HIV the reproduction number $R_{\mathrm{ehc}}\;\text{is}\;0.916\;\left(91.6\%\right)$ , also if we test (0.6) 60% of the individual who are co-infected of their HIV first the $\;R_{\mathrm{ehc}}\;$ reduces to 0.321 (32.1%). This shows that the more we test, the lower the risk of transmitting HIV and HCV.

In Figure 15, the effect of treatment and condom use on HCV reproduction numbers for the HCV model was shown on a contour plot. From the plot, if the treatment rate, r is 100% and the use of condoms is 90% it means that the reproduction number of HCV, $\;R_{\mathrm{ec}}=0.0313$ . Likewise, if 57% of the population is treated and 77% of the population use condoms $R_{\mathrm{ec}}$ will be $R_{\mathrm{ec}}=0.0626\;$ compared to when 0.7% of the HCV infected population is treated while 10.4% used the condom then $R_{\mathrm{ec}}$ rises to 0.250. This implies that to reduce the incidence of HCV transmission by the values of reproduction number, there is a need for more successful treatment where people attain SVR and avoid risk factors such as unprotected sex by use of condom, drinking, and multiple sexual partners which can make them re-infected. In Figure 16, the impact of the HCV reproduction number on the HIV reproduction number for system ( 4) it is seen that when 20% of the population is infected with HCV, 9% of the population is been infected with HIV, then the reproduction number of the co-infection, $R_{\mathrm{ech}}$ will be 0.0864 (8.64%). In the same manner, if we repeat 20% of the HCV population and 20% of HIV then we have $R_{\mathrm{ech}}$ to be 0.201 (20.1%). This simply means that as the reproduction number for HCV, $R_{\mathrm{ec}}$ increase it, in turn, increase the reproduction number of HIV $R_{\mathrm{eh}}$ . Similarly, in Figure 17, the Impact of HIV reproduction number on HCV reproduction number is represented by a contour plot. Just as seen in Figure 16. When we have 10% of the HIV population, there are 8.1% of the HCV population and the co-infection $R_{\mathrm{ech}}$ is 0.0861 (8.61%), Also, when 20% of the HIV are in the population and 2.73%of the HCV in the population, therefore we have $R_{\mathrm{ech}}$ to be 0.201 (20.1%). This also means that as HIV increase in the population, HCV also increase. This simply implies that to control HCV, HIV cases will be reduced which is attributed to the same transmission process and it is vice versa. Hence to ensure the extinction of the co-infection in the population, if HCV is reduced it will in turn impact HIV and together if the two viruses $R_{\mathrm{ec}}\;\text{and}\;R_{\mathrm{eh}}$ are low then there will be a reduction in the co-infection reproduction number, $R_{\mathrm{ech}}.$

## Conclusion

In this study, we developed and studied a mathematical model for the dynamical behavior of both HIV/AIDS and HCV co-infection, which incorporates therapy for the two diseases, vertical transmission in HIV cases, awareness and unawareness of HIV infection, inefficient follow-up of HIV on treatment, and efficient condom use.

The stability analysis of the endemic equilibria revealed that: whenever the reproduction number is less than one, the unique disease-free equilibrium is both locally and globally asymptotically stable. Also, whenever the reproduction number is greater than one, the HCV-free endemic equilibrium is both globally and locally asymptotically stable. The examination of reproduction rates indicates that HCV treatment has a positive effect on HCV and HIV-HCV co-infection reduction.

The results suggest that policymakers should consider specific measures to minimize HIV infection, such as: developing campaigns to warn individuals about the consequences of having multiple sexual partners; distributing more condoms to individuals; continuing treatment for chronic HCV and AIDS and pursuing the inquiry of new and better drugs to combat HIV; treating infected newborns with HIV and advising pregnant women about the advantages of HIV counseling and testing, treatment; and treating newborns infected with HIV. Regarding HCV infection, therapy and other measures (e.g., greater promotional awareness about the disease and its transmission methods, among others) are highly suggested so as to achieve reduction in the number of chronic carriers and infectious.

Despite the fact that this outcome is purely determined by the parameter values, it nevertheless implies that greater HCV transmission fuels HIV/AIDS and its development, hence playing a key part in the latter's increasing widespread. The same may be said for the influence of HIV/AIDS on HCV, as both HIV/HCV diseases exacerbate one another. Thus, treatment of HCV cases in areas with high HIV/AIDS prevalence will mitigate the impacts of HCV on HIV/AIDS epidemics and vice versa. Simulations indicate that the treatment of HCV has the potential to significantly minimize the detrimental result of HCV on HIV/AIDS epidemics.

Therefore, it is possible to reduce the burden produced by HIV and HCV infection and their co-morbidity.

Future research will investigate the impact of needle sharing on HIV and HCV transmission rates, as well as the application of the model to actual Portuguese data and calculation of its parameters.

## Data availability

Data used in this research can be found in Table 2: Parameters used in the numerical simulations of model.

### Extended data

Zenodo: OE-Abiodun/HIV-HCV-COINFECTION-SIM-CODE: HIV-HCV Co-infection https://doi.org/10.5281/zenodo.7373739. ^57^

This project contains the following extended data:

• F1000HIV-HCV APPENDICES.docx. Appendix A- A full model endemic equilibrium point result with *k*_1_ − *k*_13_ *and* *A*_1_ − *A*_31_ values. Appendix B- Proof of the global asymptotically stable (GAS) of disease-free equilibrium for the full model.

## Software availability

Source code available from: https://github.com/OE-Abiodun/HIV-HCV-COINFECTION-SIM-CODE/releases/tag/v3.0.0.

Archived source code at time of publication: https://doi.org/10.5281/zenodo.7373739. ^57^

License: GPL-3.0 license
