# Supplementary material for: Mathematical modeling of HIV-HCV co-infection model: Impact of parameters on reproduction number
Source: F1000Res. 2022 Dec 19;11:1153. Originally published 2022 Oct 10. [Version 2] doi: 10.12688/f1000research.124555.2 (PMC9817180; doi:10.12688/f1000research.124555.2)
Supplement: Peer review history

# Peer review: Reviewer response for version 2

**DOI:** 10.5256/f1000research.141544.r158425

**Published:** 2022-12-21

**Authors:** Afeez Abidemi

**Affiliations:** 1 Department of Mathematical Sciences, Federal University of Technology, Akure, Nigeria

The authors have addressed all the comments raised.

Is the work clearly and accurately presented and does it cite the current literature?

Yes

If applicable, is the statistical analysis and its interpretation appropriate?

Not applicable

Are all the source data underlying the results available to ensure full reproducibility?

Yes

Is the study design appropriate and is the work technically sound?

Yes

Are the conclusions drawn adequately supported by the results?

Yes

Are sufficient details of methods and analysis provided to allow replication by others?

Yes

Reviewer Expertise:

Mathematical modelling, mathematical biology, optimal control theory, nonlinear dynamics, population dynamics

I confirm that I have read this submission and believe that I have an appropriate level of expertise to confirm that it is of an acceptable scientific standard.

# Peer review: Reviewer response for version 2

**DOI:** 10.5256/f1000research.141544.r158426

**Published:** 2022-12-20

**Authors:** Adewale F. Lukman

**Affiliations:** 1 Department of Epidemiology and Biostatistics, University of Medical Sciences, Ondo City, Nigeria

I am satisfied with the revision. I recommend full approval for the manuscript.

Is the work clearly and accurately presented and does it cite the current literature?

Yes

If applicable, is the statistical analysis and its interpretation appropriate?

Not applicable

Are all the source data underlying the results available to ensure full reproducibility?

Yes

Is the study design appropriate and is the work technically sound?

Yes

Are the conclusions drawn adequately supported by the results?

Yes

Are sufficient details of methods and analysis provided to allow replication by others?

Yes

Reviewer Expertise:

Biostatistics

I confirm that I have read this submission and believe that I have an appropriate level of expertise to confirm that it is of an acceptable scientific standard.

# Peer review: Reviewer response for version 1

**DOI:** 10.5256/f1000research.136759.r153204

**Published:** 2022-11-21

**Authors:** Afeez Abidemi

**Affiliations:** 1 Department of Mathematical Sciences, Federal University of Technology, Akure, Nigeria

In this paper, the authors developed and rigorously analysed a compartmental mathematical model describing the dynamics of Human Immunodeficiency Virus (HIV) and Hepatitis C Virus (HCV) co-infection. I have the following comments/observations and questions on the paper:

1. The overall command of English seems good, however there are several grammatical errors all over the paper. For instance, in the abstract, “Hepatitis C Virus (HCV) and Human Immunodeficiency Virus (HIV) are both as classified blood-borne viruses since they are transmitted through contact with contaminated blood” is not grammatically correct. There are many incomplete sentences as well. For example, the phrase “and antiretroviral drugs are more harmful to the.” as appeared in the seventh line of the second paragraph under introduction section is incomplete. It is difficult to read the meaning of some sentences. An example is the sentence “Examining prevention, diagnosis, screening, HIV knowledge and awareness, condom use, and largely using numerical simulations, ignorance and awareness, and condom use and mostly employs numerical simulations”. Therefore, it is strongly advised and it is the authors’ responsibility to recheck the whole manuscript and fix all the grammatical errors. Moreover, in the entire manuscript, insert (,) and (.) wherever required in the equations.
2. There are no definitions for some acronyms; PLHIV, HBV, ODE, HAART, ART. The authors should ensure that they include the meaning of each of the acronyms at its first mentioning.
3. Under full model description, notations for the AIDS populace aware and on treatment and HIV-positive individuals in stage-IV co-infected with chronic HCV as given in the description ( *A _A_* ( *t*) *, A _AC_* ( *t*) *)* are different from what later appeared in the system Eq. (1) ( *A _a _(* *t*), *A _aC _* ( *t*)). The authors should correct this.
4. It is difficult to translate the flow diagram into the model equations in system (4). The authors should improve on the quality of the flow diagram of the proposed model.
5. The notation for AIDS-induced death rate given before Equation (2) does not align with the used notation ( *d _a_*) in the model equations. Please, correct it.
6. “( *r _i_, i = *1, 2, *...*)” should not be enclosed in a bracket. More so, is index *i* uncountable? Please be specific about all the integer-indexed rates *r _i_, θ _i_, δ _i _* throughout the manuscript. For instance, “ *r _i_*, *i* = 1, 2, ...” should be *r _i_*, *i* = 1, 2, ..., 5  according to system (4). In Table 1, α _1_ should be α *_i_*.
7. The statement “Mathematically, the flow chart leads to the 15 systems of ordinary differential equations listed below:” immediately before model (4) should be “Mathematically, the flow chart leads to the system of 15 ordinary differential equations given by”.
8. “the system dynamics (3.4)” immediately before Eq. (6) should be “the system dynamics (4)”.
9. It is wrong to say that "The system variable (1) are positive ..." in Theorem 1 because (1) refers to the total population. Please, correct this accordingly.
10. In the proof of Theorem 1, the statement “It follows from the model's first equation (1) that” is misleading. The authors should make the right equation reference. In general, the authors should check the proof of Theorem 1. There are many mistakes. For instance, what is the meaning of “d/d *t* = ...”? The exponents are wrongly written.  Take for instance, " *e*[]" should be *"e* ^[]^". Lastly, how the authors arrived at the conclusion *S* ≥ 0 from the preceding expression is not clear. Please explain.
11. What are R0' and RN as mentioned immediately under section "Disease-free equilibrium and the effective reproduction number"? Please, give their meanings.
12. DFE of the full model (4) is *E*_0_ *_HC_* not *E _oC_*. Correct accordingly.
13. According to the authors, Theorem 2 should be Lemma 2. Please, make correction appropriately.
14. What is “E c” in Theorem 3?
15. What are the variables *x*_1_, *x*_2_, *x*_3_ that appear in the Jacobian matrix (16)? Are the *trace* < 0 and *determinant *> 0  always true for the Jacobian matrix? The authors should mention the conditions under which the trace is non-positive and determinant is positive.
16. The statement “… and the right side of the second equation is differentiated with regards to *C _C_*” immediately before Eq. (19) should be “… and the right side of the third equation is differentiated with regards to *C _C_*”.
17. What is the physical interpretation of the result in Eq. (22)? The authors should add this result.
18. What about the endemic equilibrium of the full system (4)? The authors should say something about it.
19. There is no section 2.2.1 as mentioned immediately before Eq. (23). The authors should correct this.
20. In Lemma 5, “submodel (9)” should be “submodel (12)”. Please, ensure this correction for the rest of this part.
21. Disease-free equilibrium *E*_0 _is undefined throughout the manuscript. The authors should fix this.
22. “GSA” as mentioned immediately before the numerical simulation section should be “GAS”.
23. Section 2.2.3 in the caption of Figure 4, Theorem 3.12 in the caption of Figure 5.
24. In “conclusion section”, the last sentence should be merged with the paragraph before the paragraph that highlights the direction of future studies.

Is the work clearly and accurately presented and does it cite the current literature?

Yes

If applicable, is the statistical analysis and its interpretation appropriate?

Not applicable

Are all the source data underlying the results available to ensure full reproducibility?

Yes

Is the study design appropriate and is the work technically sound?

Yes

Are the conclusions drawn adequately supported by the results?

Yes

Are sufficient details of methods and analysis provided to allow replication by others?

Yes

Reviewer Expertise:

Mathematical modelling, mathematical biology, optimal control theory, nonlinear dynamics, population dynamics

I confirm that I have read this submission and believe that I have an appropriate level of expertise to confirm that it is of an acceptable scientific standard, however I have significant reservations, as outlined above.

# Response

**Published:** 2022-11-24

**Authors:** Oluwakemi Abiodun

**Affiliations:** Landmark University, Nigeria

Dear Reviewer, Dr Afeez Abidemi.

 Thank you for your comments and recommendations.

 All comments, observations and recommendations from comment number 1 to 23 have been addressed.

 For comment number 11, R _0  _is the reproduction number while the RN is a typo which has been deleted.

 For comment number 14, E _c_^* ^is the endemic equilibrium point for the Hepatitis C virus (HCV)

 In comment 15, xi, x2, x3 are representation of the state variables

 Furthermore, for *trace <* 0  and *determinant *> 0,  they are not always true for the Jacobian matrix. They are true under the condition for DFE and Endemic equilibrium (EE) whenever R _0 _< 1 and if R _0 _> 1 respectively.

 Physical interpretation of Eqn (22) now (23) mentioned in comment 17 has been included in the new version submitted.

 Thank you.

# Peer review: Reviewer response for version 1

**DOI:** 10.5256/f1000research.136759.r153205

**Published:** 2022-11-08

**Authors:** Adewale F. Lukman

**Affiliations:** 1 Department of Epidemiology and Biostatistics, University of Medical Sciences, Ondo City, Nigeria

The idea presented in the article is innovative and well-written. I recommend acceptance for indexing subject to minor revision.

 The authors developed and investigated a mathematical model for the dynamical behavior of HIV/AIDS and HCV co-infection, which includes therapy for both diseases, vertical transmission in HIV cases, unawareness and awareness of HIV infection, inefficient HIV treatment follow-up, and efficient condom use. The Methods and theorem in the articles are well stated and clear enough for future work purposes.

 The article recommends that HCV should be treated first in those co-infected with HIV. This is a strong recommendation for the public health sectors to take note of in reducing the increasing rate of viral load in HIV patients. The authors should consider adding those inferred steps to the conclusion part of the abstract session for easy access for the reader.

 The problem is addressed with a detailed numerical simulation. Conclusively, the authors should consider extending the work by adding controls on the model to know what control parameters will be recommended to control the menace of the co-infection.

 The authors should go through the grammatical structure of the manuscript. For instance, I made a few revisions to the Abstract Background:

 "Hepatitis C Virus (HCV) and Human Immunodeficiency Virus (HIV) are classified as blood-borne viruses since they are transmittable through contact with contaminated blood. ****Approximately 1.3 million of the 2.75 million global HIV/HCV carriers inject drugs (PWID). HIV co-infection **harms** the progression of HCV, resulting in **higher **rates of HCV persistence after acute infection, higher viral levels, and accelerated progression of liver fibrosis and end-stage liver disease."

Is the work clearly and accurately presented and does it cite the current literature?

Yes

If applicable, is the statistical analysis and its interpretation appropriate?

Not applicable

Are all the source data underlying the results available to ensure full reproducibility?

Yes

Is the study design appropriate and is the work technically sound?

Yes

Are the conclusions drawn adequately supported by the results?

Yes

Are sufficient details of methods and analysis provided to allow replication by others?

Yes

Reviewer Expertise:

Biostatistics

I confirm that I have read this submission and believe that I have an appropriate level of expertise to confirm that it is of an acceptable scientific standard.
